# The iRhom homology domain is indispensable for ADAM17-mediated TNFα and EGF receptor ligand release

**DOI:** 10.1007/s00018-021-03845-3

**Published:** 2021-05-05

**Authors:** Stefan Düsterhöft, Selcan Kahveci-Türköz, Justyna Wozniak, Anke Seifert, Petr Kasparek, Henrike Ohm, Shixin Liu, Jana Kopkanova, Juliane Lokau, Christoph Garbers, Christian Preisinger, Radislav Sedlacek, Matthew Freeman, Andreas Ludwig

**Affiliations:** 1 Institute of Molecular Pharmacology, Medical Faculty, RWTH Aachen University, Wendlingweg 2, 52074 Aachen, Germany; 2 Czech Centre for Phenogenomics, Institute of Molecular Genetics of the Czech Academy of Sciences, Prague, Czech Republic; 3 Department of Pathology, Medical Faculty, Otto Von Guericke University Magdeburg, Magdeburg, Germany; 4 Proteomics Facility, IZKF, RWTH Aachen University, Aachen, Germany; 5 Sir William Dunn School of Pathology, University of Oxford, Oxford, UK

**Keywords:** iRhom, ADAM17, Ectodomain shedding, TNF, Growth factors, iRhom homology domain

## Abstract

Membrane-tethered signalling proteins such as TNFα and many EGF receptor ligands undergo shedding by the metalloproteinase ADAM17 to get released. The pseudoproteases iRhom1 and iRhom2 are important for the transport, maturation and activity of ADAM17. Yet, the structural and functional requirements to promote the transport of the iRhom-ADAM17 complex have not yet been thoroughly investigated. Utilising in silico and in vitro methods, we here map the conserved iRhom homology domain (IRHD) and provide first insights into its structure and function. By focusing on iRhom2, we identified different structural and functional factors within the IRHD. We found that the structural integrity of the IRHD is a key factor for ADAM17 binding. In addition, we identified a highly conserved motif within an unstructured region of the IRHD, that, when mutated, restricts the transport of the iRhom-ADAM17 complex through the secretory pathway in in vitro, ex vivo and in vivo systems and also increases the half-life of iRhom2 and ADAM17. Furthermore, the disruption of this IRHD motif was also reflected by changes in the yet undescribed interaction profile of iRhom2 with proteins involved in intracellular vesicle transport. Overall, we provide the first insights into the forward trafficking of iRhoms which is critical for TNFα and EGF receptor signalling.

**Supplementary Information:**

The online version contains supplementary material available at 10.1007/s00018-021-03845-3.

## Introduction

Maintaining balanced regulation of cell-to-cell communication is the basis of multicellularity. One way to generate signals is proteolytic release of biologically active ectodomains from membrane-tethered proteins, which is also known as ectodomain shedding. Here, a disintegrin and metalloproteinases (ADAM)17 plays a crucial role in the regulation of many signalling pathways such as in developmental and regenerative processes by releasing ligands of the epidermal growth factor receptor (EGFR) [2, 3]. Systemic absence of ADAM17 activity in mice causes developmental defects and lethality [3]. Furthermore, ADAM17 is instrumental in the inflammatory response. This includes the release of tumour necrosis factor alpha (TNFα) [4, 5] and its receptors as well as the interleukin 6 (IL6) receptor [6–8]. Unsurprisingly, dysregulation of ADAM17 activity is implicated in pathologies such as chronic inflammation and cancer progression [7–10].

To date, different levels of regulating ADAM17 are known. One basic control of its activity is achieved by controlling ADAM17 localisation. ADAM17 is synthesised into the ER as a proform (immature ADAM17) with an inhibitory prodomain and undergoes maturation by furin-like proteases in the Golgi [11, 12]. Recently, iRhoms, which are pseudoproteases within the superfamily of rhomboid proteases [13, 14], have been found to be indispensable for the ER exit of ADAM17 [15–18]. Yet, it is still an enigma how iRhoms promote the ER to Golgi transport of ADAM17, which is necessary for subsequent ADAM17-dependent signalling.

Two iRhom proteins exist in mammals, namely iRhom1 (RHBDF1) and iRhom2 (RHBDF2). While iRhom1 is present in most cells, iRhom2 seems to be predominantly expressed in immune cells. This is in line with the findings in iRhom2 deficient mice, which do not have an obvious phenotype. Yet, when challenged with pro-inflammatory stimuli, they show an impaired immune response, which is evident from impaired TNFα production [15, 16]. In contrast, mice deficient in iRhom1 or both iRhoms show developmental phenotypes [17, 18]. Although both iRhoms differ in some regions, their overall topology is the same. They consist of a rhomboid core of seven transmembrane helices, which is characteristic for all rhomboids. However, in contrast to the proteolytically active rhomboids the active site is extinguished in both iRhoms. The first helix of the rhomboid core was implicated to be responsible for the interaction between iRhoms and ADAM17 [19, 20]. In addition, iRhoms possess two other structural units: a long cytosolic N-terminus and a large luminal region between transmembrane helix (TMH) 1 and TMH2 of the rhomboid core called IRHD (iRhom homology domain) [13, 15]. The N-terminus is reported to be crucial for the ADAM17 shedding activity on the cell surface due to its phosphorylation [20, 21] as well as for the stability of the iRhom-ADAM17 complex by binding the iRhom interactor FRMD8 [22, 23]. The IRHD together with the rhomboid core are the evolutionary highest conserved parts of iRhoms. However, while the rhomboid core is the common feature of all members in the rhomboid family, the IRHD is unique for iRhoms not only among rhomboids but also among all known structural domains. To date, neither structure nor function of the IRHD is known.

In this study we analysed the IRHD in silico, in vitro and in vivo. We found that the integrity of the whole IRHD is needed for efficient ADAM17 interaction. Furthermore, we identified a highly conserved motif within the IRHD, which is indispensable for ER to Golgi transport of iRhoms. This motif is located within a non-structured stretch of the IRHD. We here provide multiple lines of evidence that the integrity of this motif is crucial for the function of human as well as murine iRhom2 in vitro, ex vivo and in vivo. A single-point mutation within this motif is sufficient to abrogate the ER to Golgi transport of iRhom2 without affecting neither the folding of the IRHD nor binding to ADAM17. In contrast, mutations within this motif drastically increased the half-life of iRhom2 and bound ADAM17. However, since the forward trafficking of the iRhom2-ADAM17 complex is negatively affected by mutations within this motif, neither ADAM17 maturation nor ADAM17-mediated shedding can take place.

We furthermore for the first time identified novel iRhom2 interaction partners, which are critically involved in intracellular vesicle trafficking. Several proteins belonging to the family of the soluble NSF attachment proteins receptors (SNARE), which are crucial for intracellular vesicle transport especially vesicle fusion, differently interact with iRhom2 throughout the secretory pathway.

## Materials and methods

### In silico* analysis*

Sequence alignments were performed with the webtool Clustal Omega [24]. These alignments were used to generate a secondary structure prediction as well as a score for the conservation utilising the webtool PRALINE [25] and Jpred4 [26]. Parts of the alignments are also depicted as weblogo presentation which were created by utilising the webtool WebLogo 3[27]. The iRhom protein sequences (iRhom1 and iRhom2) of the following species were used: *Alligator mississippiensis* (american alligator), *Antrostomus carolinensis (*Chuck-will's-widow), *Ascaris suum* (pig roundworm), *Caenorhabditis elegans* (roundworm), *Camponotus floridanus (*florida carpenter ant), *Danio rerio (*zebrafish), *Daphnia magna (*water flea), *Dracunculus medinensis (*guinea worm), *Drosophila melanogaster (*fruit fly), *Gallus gallus (*chicken), *Homo sapiens (*human), *Ixodes scapularis (*black-legged tick), *Larimichthys crocea (*large yellow croaker), *Latimeria chalumnae (*west Indian ocean coelacanth), *Lygus Hesperus* (western plant bug), *Macaca fascicularis (*crab-eating macaque), *Macaca mulatta* (rhesus macaque), *Oreochromis niloticus (*Nile tilapia), *Poecilia formosa (*Amazon molly), *Pongo abelii* (Sumatran orang-utan), *Trichinella britovi (*parasitic roundworm), *Trichinella spiralis (*trichina worm), *Tupaia chinensis (*Chinese tree shrew), and *Xenopus tropicalis* (western clawed frog). If two iRhom forms were available/exist, both were used.

### Cloning

To insert different constructs into a vector the NEBuilder HiFi DNA assembly master mix (NEB, E2621L) was used according to the manufacturer’s manual. All iRhom constructs were cloned into the pMOWS backbone [28] and thereby also fused to a 3xHA tag. Either a plasmid with puromycin resistance (pMOWS_Puro) or zeocin resistance (pMOWS_Zeo) was used. Site-directed mutagenesis was performed by using overlapping PCR [29]. To design miR2_ΔIRHD the IRHD was replaced by GS-linker sequences (GSSGGSG-EQKLISEEDL-GSGSSGG). To design miR2_IRHD1 and miR2_IRHD2 the IRHD was divided in a way to ensure an even number of cysteine residues in both halves. The respective deletion within both constructs was also replaced by a GS-linker.

### Cell culture

HEK293 cells and MEFs, double deficient for iRhom1 and iRhom2 (MEF_dKO), were cultured in a humidified incubator at 37 °C with 5% CO_2_ in DMEM10%. DMEM10% contains DMEM high-glucose (Sigma-Aldrich) supplemented with 10% foetal calf serum (PanBiotech), 100 mg/l streptomycin (Sigma-Aldrich) and 60 mg/l penicillin (Sigma-Aldrich). To generate cells stably expressing the protein of interest the pMOWS-vector system [28] in combination with Phoenix ampho cells, a virus-production cell line (HEK293 cell derivate; ATCC: CRL-3213) was utilised to produce viruses for viral transduction of target cells. For transfection the reagent jetPEI (Polyplus; 101-10 N) was used in accordance with the manufacturer’s manual. 6 × 10^6^ Phoenix ampho cells were transfected with 10 µg of either pMOWS_Puro or pMOWS_Zeo containing the insert of interest. 24 h later transfection medium was exchanged with 5 ml fresh DMEM10%. After additional 24 h, the virus-containing supernatant (5 ml) was harvested and mixed with 3 ml fresh DMEM10% as well as with 8 µg/µl (final concentration) polybrene (Sigma; TR-1003-G). The mixture was incubated for 15 min at room temperature and added to 1 × 10^6^ HEK293 or MEF_dKOs. After 24 h, positively transduced cells were selected with either 0.5 µg/µl puromycin (InvivoGen; ant-pr-1) or 100 µg/µl zeocin (InvivoGen; ant-zn-1) depending on the used vector. The generation and characterisation of MEFs from the indicated mouse lines has been described before [17]. BMDMs were freshly isolated from femur and tibiae of respective mouse lines as described previously [30]). HEK293 cells were obtained from German Collection of Microorganisms and Cell Cultures (GmbH DSMZ- No. ACC 305).

### Co-immunoprecipitation and enrichment of glycosylated proteins

For precipitation experiments, 8.0 × 10^6^ cells either stably expressing HA-tagged iRhom constructs or GFP (negative control) were lysed in 1 ml lysis buffer (50 mM Tris; 137 mM NaCl; 2 mM EDTA; 10 mM 1,10-Phenanthroline; pH7.5) supplemented with cOmplete™ protease inhibitor cocktail (Sigma; 11,697,498,001). When indicated cells were transfected with either human STX6 (RC202951; OriGene Technologies), human STX10 (RC215143; OriGene Technologies), human CANX (RC200229; OriGene Technologies) and mouse SEC22a (MR217787; OriGene Technologies). All of the additionally transfected constructs were myc tagged (pCMV6-Entry). The cell lysates were cleared by centrifugation at 16,000 × g for 15 min at 4 °C. 450 µl cleared lysate was used for enrichment of glycosylated proteins with 30 µl Concanavalin A sepharose (Sigma; C9017) or co-immunoprecipitations (co-IPs) with 10 µl anti-HA magnetic beads (ThermoFisher; 88,836). The respective beads were incubated with the lysate for 90 min and afterwards washed for five times with lysis buffer. After lysis buffer removal beads were prepared for analysing via western blot by adding 40 µl reducing loading buffer (3% (w/v) SDS, 16% glycerol, 8% 2-mercaptoethanol, 0.01% (w/v) bromphenol blue, 0.1 M Tris HCl, pH 6.8) to the beads and heating them at 65 °C for 20 min. Alternatively, co-IPs were used for mass spectrometry measurements.

### Deglycosylation

For deglycosylation experiments IPs were performed as described above. After the last washing step, the beads were incubated with either the glycosidase endoglycosidase H (Endo H) (NEB; P0702S) or with the glycosidase N-glycosidase F (PNGase F) (NEB; P0704S) according to the manual of the manufacturer except for the initial denaturation step, which was carried out at 60 °C.

### Western blotting

Samples were separated by SDS-PAGE and transferred onto polyvinylidene difluoride (PVDF) membranes (Millipore, Immobilon-FL). Afterwards, membranes were blocked with 5% (w/v) non-fat dry milk in TBS (50 mM Tris, 150 mM NaCl, pH 7.4) for 20 min at room temperature, and subsequently incubated with primary antibodies in 0.1% Tween-TBS and 1% (w/v) BSA overnight at 4 °C. After three washing steps with 0.1% Tween-TBS the membrane was incubated with fluorophore-coupled secondary antibody for 1 h at room temperature. After additional washing steps once with 0.1% Tween-TBS and three times with TBS, stained proteins were detected via the Odyssey 9120 imager system (LI-COR) and the ChemiDoc MP Imaging System (Bio-Rad). For quantification, the band intensities were measured with the software Image Studio Lite (LI-COR). Where indicated, the 4–15% Mini-PROTEAN TGX stain-free precast protein gels (Bio-Rad, 4568083) were used. The following primary antibodies with the indicated dilutions were used: αADAM17 (1:1000; Abcam; ab39162), αHA (1:2000; Biolegend; 901502), αTransferrin-receptor (1:2000; Abcam; ab1086), αiRhom2 (1:2000; Sigma; SAB1304414). The following secondary antibodies with the indicated dilutions were used: DyLight-680-conjugated αmouse (1:20000; Thermo; 35519), DyLight-800-conjugated αrabbit (1:20000; Thermo; 35571), horse radish peroxidase (HRP)-conjugated goat anti-mouse and anti-rabbit (1:20000; Jackson ImmunoResearch Laboratories, Inc).

### Shedding activity—AP-assay

Shedding activity of ADAM17 was measured by an alkaline phosphatase (AP)–based assay. For this shedding activity assay 5 × 10^6^ cells were seeded in a 10-cm dish and transiently transfected with the ADAM17 substrate fused to an alkaline phosphatase (AP). Transient transfection was performed via the use of Lipofectamine 3000 (Thermo; L3000015). After 24 h 2 × 10^5^ cells/well were transferred into 24-well plates. 24 h later cells were stimulated with 100 nM PMA (Sigma; P1585) or 30 µM TRAP6 (Bachem; H-2936) or left unstimulated (treated with vehicle DMSO). The ADAM17 inhibitor TAPI1 (Sigma; SML0739) was used as control. Cells were incubated for 120 min at 37 °C. The shedding activity was assessed by measuring the AP activity in the supernatant and in cell lysates (lysis buffer: 50 mM Tris; 137 mM NaCl; 2 mM EDTA; 10 mM 1,10-Phenanthroline; pH7.5). By adding p-Nitrophenyl phosphate (PNPP) solution (Thermo; 37620) the AP activity could be continuously measured at 405 nm with the FLUOstar Optima (BMG LABTECH). To assess the AP activity the slope (change of absorption at 405 nm per min) was calculated. The amount of ADAM17 activity was calculated as PNPP substrate turnover (AP activity) in the supernatant in relation to the total turnover in supernatant plus cell lysate. The following ADAM17 substrates cloned into pCDNA3.1 were used: AP-IL1R_II_, AP-HBEGF, AP-TGFα, AP-AREG, AP-IL6R.

### Flow cytometric analysis

PBS supplemented with 0.2% BSA was used as assay buffer and all steps of the staining process were performed at 4 °C or on ice. 2 × 10^5^ cells of interest were stained with primary antibody for 1 h. Afterwards the cells were washed two times with 400 μl assay buffer. The secondary antibody was added and the cells incubated in the dark for 45 min. After two additional washing steps the fluorescence signal was analysed by flow cytometry (LSRFortessa, BD Biosciences, Heidelberg, Germany) and evaluated with FlowJo V10 software. To identify the cell surface expression the geometric mean of the fluorescence intensity was determined. The following primary antibodies with the indicated dilutions were used: αADAM17 (1:100; R&D Systems; MAB 9301), αHA (1:500; Biolegend; 901502), αADAM10 (1:100; R&D, MAB946). The following secondary antibodies with the indicated dilutions were used: allophycocyanin-conjugated αmouse (1:200; Jackson ImmunoResearch; 115–135-164), phycoerythrin-conjugated αrat (1:100; Jackson ImmunoResearch; 112–116-071).

### Cycloheximide-based pulse chase experiment

2 × 10^6^ HEK293 cells or MEF_dKOs overexpressing the indicated iRhom constructs were seeded. 24 h later the cells were treated with 10 µg/ml cycloheximide (CHX) for the indicated time frames. Cells were harvested and counted again to adjust all samples to an equal cell count. Harvested cell samples were lysed and used for co-IP and enrichment of glycosylated proteins as described before.

### Lysosomal inhibition

3 × 10^6^ HEK293 cells or MEF_dKOs overexpressing the indicated iRhom constructs were seeded. 24 h later the cells were treated without 500 nM bafilomycin A1 (Sigma) or vehicle control (DMSO) for the indicated time frames (4 or 8 h). Cells were harvested and lysed and used for co-IP or enrichment of glycosylated proteins as described before.

### Density gradient centrifugation

HEK293 cells overexpressing the indicated iRhom constructs with 90% confluency (in 10-cm dish) were harvested. Cell pellets were resuspended in 1 ml lysis buffer (50 mM Tris; 137 mM NaCl; 2 mM EDTA; 10 mM 1,10-Phenanthroline; pH7.5, DNAse) and lysed via bead milling. The supernatant/lysate was cleared by centrifugation (4 °C) for 10 min at 1000 g and for 10 min at 3000 g. Density gradient was prepared as follows: A working solution was prepared by mixing 5 vol. of OptiPrep (Sigma; D1556-250ML) with 1 vol. of diluent solution (0.25 M sucrose; 6 mM EDTA, 60 mM HEPES–NaOH) to get a 50% iodixanol solution. Discontinuous iodixanol gradient is produced by mixing working solution with homogenization medium (0,25 M sucrose; 1 mM EDTA; 10 mM Hepes–NaOH) to get the following iodixanol concentrations: 2.5% (1 ml); 5.0% (1.5 ml); 7.5% (0.5 ml); 10.0% (0.5 ml); 12.5% (0.5 ml); 15.0% (1.5 ml); 17.5% (0.5 ml); 20.0% (0.5 ml); 25.0% (0.5 ml) and 30% (0.5 ml). Discontinuous density gradient was formed by layering the solutions with the different iodixanol concentrations in a centrifugation tube on ice. Cleared cell lysate was added to the discontinuous density gradient and centrifuged at 200,000×g for 2 h 15 min (4 °C). After centrifugation the gradient is carefully separated into eight fractions (1.1 ml each). Fractions are centrifuged at 100,000×g for 40 min (4 °C) and pellets are resuspended in 40 µl lysis buffer and 10 µl 5 × reducing loading buffer. Samples were heated at 65 °C for 20 min for western blotting.

### Mass spectrometry analysis

Co-immunoprecipitation experiments (*n* = 3) for interactome analysis were prepared as described above, except that after enrichment the beads were washed three times with lysis buffer and three times with lysis buffer w/o detergent. The dried beads from the individual IPs were then processed for MS-analysis as described previously [31]. Briefly, the beads were digested at room temperature for 1 h with 5 μg/mL Trypsin (in 2 M Urea, 50 mM Tris–HCl pH 7.5). The supernatant was transferred into a fresh tube and the beads were washed twice with 2 M urea, 50 mM Tris–HCl pH 7.5 and 1 mM DTT. The supernatants were pooled and protein digestion took place o.n. at room temperature. The digested peptides were then first modified with iodoacetamide, acidified, desalted using homemade C18-tips and finally lyophilised. The dried peptides were resuspended in 3% formic acid (FA)/5% acetonitrile (ACN) and loaded onto the nanoLC system (RSLCnano, Thermo Scientific). Peptides were trapped on a precolumn (Acclaim PepMap100, C18, 5 µm, 100 Å, 300 µm i.d. × 5 mm, Thermo Scientific) for 10 min and subsequently separated using an analytical column (Easyspray 50 cm column (ES803); Thermo Scientific) employing a 150-min gradient (0–10 min: 5% buffer B (buffer A: 0.1% FA; buffer B: 80% acetonitrile, 0.1% FA), 10–104 min: 10–35% buffer B, 104–114 min: 35–45% buffer B, 114–115 min: 45–95% buffer B, 115–119 min: 95% buffer B, 119–120 min: 95–5% buffer B, 120–150 min: 5% buffer B). The spray voltage was set to 2 kV and the capillary temperature was set to 250 °C. All samples were analysed on a Q Exactive plus mass spectrometer (Thermo Scientific) in duplicate (technical replicates) in data dependent mode. Full MS settings were the following: 70,000 resolution; AGC target, 1e6; maximum injection time, 50 ms; scan range, 350–1600 m/z. dd-MS2 settings were: 17,500 resolution; AGC target: 1e5; maximum injection time: 55 ms; top 20 precursor fragmentation; isolation window, 2.0 m/z; collision energy, 27. dd settings were: minimum AGC, 5e2; 20 s dynamic exclusion; only 2 + to 5 + peptides were allowed.

Analysis of the raw data was done using MaxQuant (version 1.6.3.3) with the built-in Andromeda search engine [32]. The spectra were searched against the human SwissProt database version 06/2018 (only reviewed and canonical sequences); the database was customised adding the mouse iRhom2 entry (RHDF2_MOUSE, UniprotID: Q80WQ6, with and without point mutation and a C-terminal 3xHA tag). The MaxQuant default settings (including the mass tolerance) were used. Specific settings were as follows: Trypsin as the protease (two missed cleavages); Carbamidomethylation (Cys) as the fixed modification; Oxidation (Met) and N-terminal protein acetylation were used as variable modifications: The false discovery rate was 0.01 on both peptide and protein level and the minimum peptide length was set to seven amino acids. Quantification was done using the label-free quantitation algorithm from MaxQuant.

The proteinGroups.txt result file (Sup. Tab. 1) derived from the MaxQuant search was further analysed using Perseus (version 1.6.10.0) [33]. The LFQ intensities from all biological and technical replicates (GFP, wt, miR2_W538S mutant, 18 columns in total) were loaded as the main columns. The protein list was filtered for reversed hits, contaminants, and “only identified by site” entries. Further requirements for protein inclusion included minimum one unique peptide and two total peptides (razor + unique). The three individual biological replicates (and technical replicates) were grouped into their corresponding experiments (GFP, wt (iR20), miR2_W538S mutant (iR2A)). Data were transformed by applying log2. Proteins were only included in the final data set, if they were identified in all replicates in at least one group (min six in one group). The remaining list was subjected to imputation of missing values based on normal distribution (Perseus default settings). The data were then further analysed using the two-sample tests option. The resulting file was used for generation of the Volcano plots (Sup. Table 1). The settings for the volcano plot were: p-value < 0.01 and a ratio of > fourfold change (> 2 (–log (10) p-value) and > 2 (log (2) ratio).

### Generation of iRhom2 mutant mice

iRhom2^W538S^ and iRhom2^Del^ mice were generated by electroporation of C57BL/6 N zygotes with Cas9 protein (IDT) (500 ng/µl), synthetic guide RNA (IDT) (20 µM, 5′ CCTCC AGTGG GGCCC ACATC 3′) and a single-stranded deoxyoligonucleotide (Sigma) (5 µM, 5′ TAGGA CCTGT GAAGA GCCTG CCTCC AGTGG GGCCC ACATC AGTCC GGATG ATATC ACCAA GTGGC CGGTG AGTAG GAGAT CCATG GAG 3′) as a repair template. Electroporation was performed using NEPA21 electroporator (Sonidel) with following pulse parameters: 40 V, 3.5msec, 50msec interval, 4 pulses, decay rate 10%, + polarity (poring pulses) and 5 V, 50msec, 50msec interval, 5 pulses, decay rate 40%, ± polarity (transfer pulses). Manipulated embryos were implanted into foster mothers at the stage of zygotes, typically 1–3 h after the electroporation. Newborn founder mice were genotyped using primers iRhom2_Fwd (5´ GACCACCTGTCCACCCTCTA 3´) and iRhom2_Rev (5´ AGGATGGGACAAGCTCCTTT 3´) followed by Sanger sequencing. iRhom2^Del^ mice possess a deletion of 13 nucleotides (Δ13: TGGGCCCCACTGG) positioned directly before iCERES resulting in an early translational stop. A list of the in experiments used mice can be found in the supplement file with the raw data.

### BMDM isolation

BMDMs were isolated out of femur and tibia of hind limbs of 8 to 10-week-old mice. Therefore, the muscle tissue was removed and the bones were stored in cold PBS throughout the whole isolation procedure. Femur and tibia were cut at both ends and flushed with ice-cold RPMI1640_FPS (RPMI1640 supplemented with 10% foetal calf serum, 100 mg/l streptomycin and 60 mg/l penicillin) using a syringe with an 18G needle. Cells were centrifuged at 300 g for 5 min at 4 °C and resuspended in culture medium (RPMI1640_FPS and 20% L929-conditioned medium) using a syringe with 26G needle. Cells were plated on two 15-cm cell culture dishes per mouse within 20 ml culture medium. After 72 h, 10 ml culture medium was added, followed by a culture medium change at day 6. At day 10, BMDMs were seeded in 12-well plates. During stimulation the BMDMs were cultured in the absence of L929-conditioned medium for 24 h. At the end of differentiation BMDMs were mainly (more than 90%) F4/80- and CD11b-positive as determined by flow cytometry. For LPS stimulation BMDMS were treated with min. 100 ng LPS for min. 8 h.

### Shedding activity in BMDMs

5 × 10^5^ BMDMs/well were seeded in 2 ml fully supplemented growth medium 24 h before stimulation. Afterwards medium was exchanged (750 µl) and cells were stimulated for 24 h with 100 ng/ml LPS from *E. coli* strain 0127:B8 (Sigma-Aldrich; L4516). Release of murine IL6, murine KC and murine TNFα to the supernatant of stimulated BMDMs was analysed using commercial ELISA Kits (R&D Systems, DuoSet) according to the manufacturer’s protocols. The read-out was executed with a FLUOstar OPTIMA (BMG-Labtech).

### Quantitative PCR analysis

mRNA levels for genes of interest were quantified by quantitative (q)PCR analysis and normalized to the mRNA level of the murine reference genes glyceraldehyde-3-phosphate dehydrogenase (mGapdh) and ribosomal protein S29 (mRps29). RNA was extracted using the RNeasy Kit (Qiagen; 74106) and quantified photometrically (NanoDrop; Peqlab). For bone marrow-derived macrophages 200 ng RNA was reverse transcribed using the PrimeScript™ RT Reagent Kit (Takara Bio Europe; RR036B). For tissue samples, 500 ng RNA was used, respectively. PCR reactions were performed using iTaq™ Universal SYBR® Green Supermix (Bio-Rad; 1725124) according to the manufacturer's protocol. All PCR reactions were run on the CFX Connect Real-Time PCR Detection System (Bio-Rad) with the following protocol: 5 min of initial denaturation at 95 °C, 45 cycles of 10 s denaturation at 95 °C, followed by 30 s annealing and 15 s amplification at 72 °C. Annealing temperatures and primer sequences are listed:

**Table:** 

Gene	Sequence	Annealing temperature
m*Adam17*	For *AAA CCA GAA CAG ACC CAA CG* Rev *GTA CGT CGA TGC AGA GCA AA*	57 °C
m*Gapdh*	For *CAT GGC CTT CCG TGT TCC TA* Rev *ACT TGG CAG GTT TCT CCA GG*	60 °C
m*Il6*	For *TGC AAG AGA CTT CCA TCC AGT TGC C* Rev *AAG CCT CCG ACT TGT GAA GTG GT*	59 °C
m*iRhom1*	For *TTC TTC ACT TAC TGG CTC AC* Rev *TTC CGA AGT ACC GAG TCC*	60 °C
m*iRhom2*	For *AGA GCG TGA AGT ACA TCC* Rev *TAA AGT CTC CGA GCA GTC C*	60 °C
m*Rps29*	For *CCT TTC TCC TCG TTG GGC* Rev *GAG CAG ACG CGG CAA*	61 °C

The efficiency of each qPCR sample was calculated with linear regression using the LinReg software (v2018.0; Dr J.M. Ruitjer; Academical Medical Centre Amsterdam; Netherlands). Relative quantification was performed with the E-Method (Tellmann, 2006) using the CFX Maestro 1.1 software (v4.1.2433.1219; Biorad).

### PCR (genomic DNA and cDNA)

Genomic DNA was isolated from tail cuts. Tissue was immersed in 100 µl buffer 1 (50 mM NaOH) and incubated for one hour at 95 °C and 800 rpm. 10 µl buffer 2 (1 M Tris; 10 mM EDTA; pH8.0) was added. Isolated genomic DNA was used for genotyping of mutant mice with the following protocol: 5 min of initial denaturation at 95 °C, 35 cycles of 30 s denaturation at 95 °C, followed by 30 s annealing at 61 °C and 90 s amplification at 72 °C. PCR reactions were performed using GoTaq® G2 Flexi DNA Polymerase (Promega; M7806) according to the manufacturer's protocol. The following primers were used: m*iRhom2_*wt for, TGG CCT GAT GAC ATT ACC AAG TGG C; m*iRhom2_*W538S for, AGT CCG GAT GAT ATC ACC AAG TGG C; m*iRhom2* rev, TCA GGG TTG AGG AAA GGC AGG AGC. cDNA from these mice was subjected to the same PCR protocol.

### Statistics

All experiments were done at least three times as indicated in the figure legends. Quantitative data are shown as mean with standard deviation (SD) calculated from at least three independent experiments, n numbers are specified in the figure legends. Statistics were conducted using the generalised mixed model analysis (PROC GLIMMIX, SAS 9.4, SAS Institute Inc., Cary, North Carolina, USA) and assumed to be from either normal, lognormal or beta distribution with the day of experiment conduction as random to assess differences in the size of treatment effects across the results. Residual analysis and the Shapiro–Wilk test were used as diagnostics. In the case of heteroscedasticity (according to the covtest statement) the degrees of freedom were adjusted by the Kenward-Roger approximation. All p-values were adjusted for multiple comparisons by the false discovery rate (FDR). p < 0.05 was considered significant with *p*: * < 0.05, ** < 0.01, *** < 0.001.

## Results

### The IRHD is crucial for iRhom functions

**Fig. 1:**
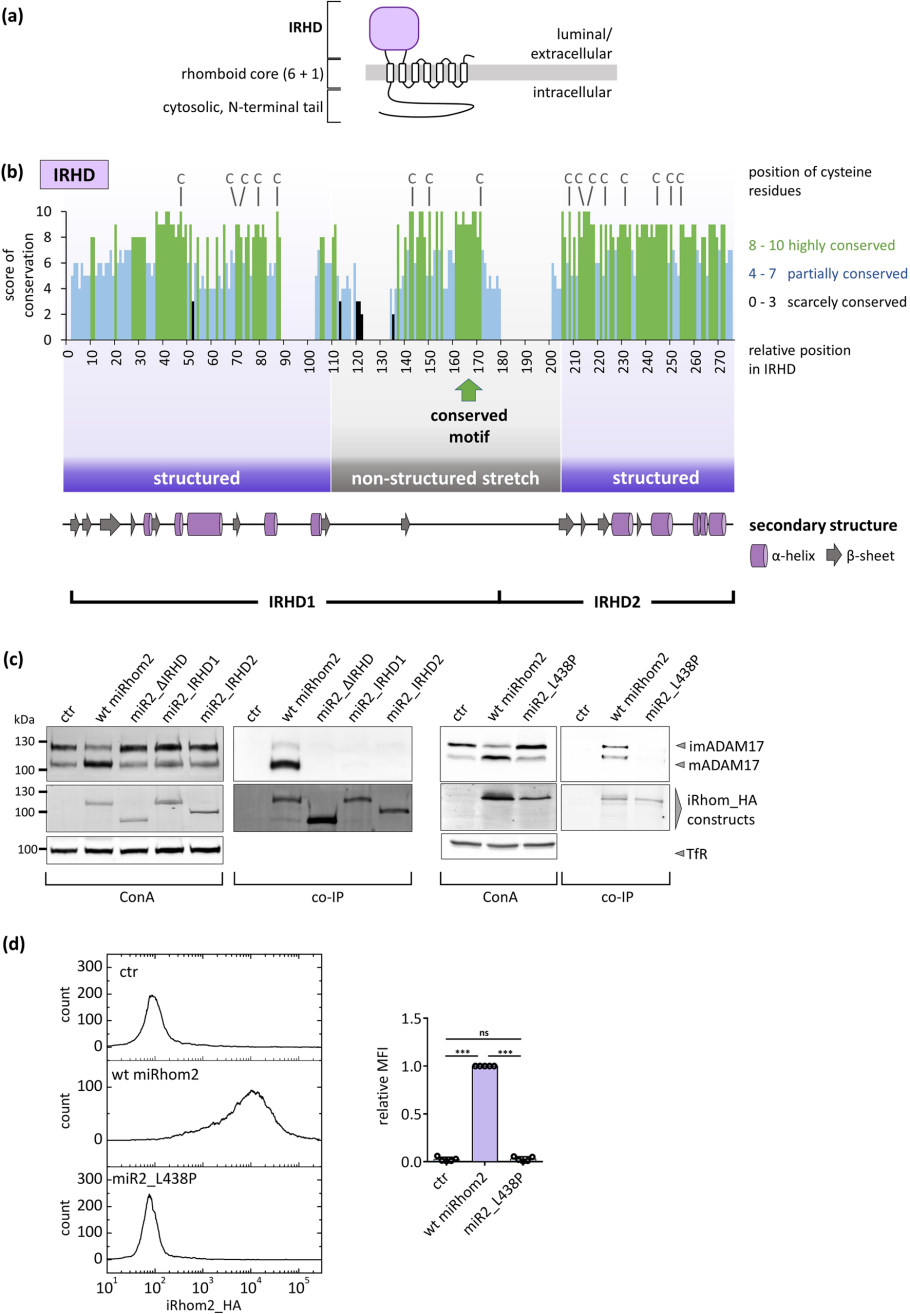
The integrity of the iRhom homology domain (IRHD) is crucial for iRhom functions. **a** Overview of the general iRhom topology. **b** The iRhom homology domains (IRHDs) of various species (iRhom1 and iRhom2) were analysed for conservation expressed as score and secondary structure. Since the IRHDs from different species have different lengths due to deletions and extensions, the numbering of the amino acid residues is relative to the hypothetical longest IRHD harbouring all extensions. The IRHD can be divided into three regions: An N-terminal and a C-terminal region, which possess secondary structures, as well as a non-structured stretch separating both structured regions. Within this non-structured stretch, a highly conserved motif can be found. Position and impact of all mutants used in this study can be found in Figure S1. **c**–**d** HEK293 cells stably expressing the indicated iRhom constructs or GFP (ctr) as negative control were utilised. HEK293 cells possess endogenous iRhoms and hence have a basal degree of ADAM17 maturation. **c** To analyse ADAM17 maturation, glycosylated proteins were enriched by concanavalin A beads (ConA). The level of ADAM17 maturation is detectable by the presence of mature ADAM17 (mADAM17) with lower molecular weight than immature (imADAM17). To analyse the binding between ADAM17 and iRhom constructs, co-IPs were performed by using the iRhom constructs (with HA tag) as bait. The transferrin receptor (TfR) served as input control. ADAM17 maturation can be evaluated by comparing the amount of immature (imADAM17) and the amount of mature ADAM17 (mADAM17). Quantitative analysis of ADAM17 maturation levels and ADAM17 binding can be found in figures S2a,b. *n* = 3. **d** Cell surface expression of iRhom constructs were measured by flow cytometry. For quantification, the geometric mean of the specific fluorescence signal was determined and normalised to the wt. *n* = 5

Although the IRHD of iRhoms represents roughly a quarter of the whole protein (Fig. 1a), neither structure nor function(s) are known. To get insights into the structure of the IRHD, a protein sequence alignment from different evolutionary distant species was used for secondary structure prediction and for scoring of conservation (Fig. 1b). Interestingly, our structural analysis predicts that the IRHD consists of two structured regions, which are separated by an unstructured stretch. As expected, structurally important positions, including the cysteine residue pattern and amino acid residues that form secondary structures, appear to be highly conserved. In contrast, the unstructured stretch contains most of the variable regions, suggesting that these positions within the IRHD sequence are actually less important for structure and possibly function. However, the unstructured stretch also contains a short portion that is highly conserved among iRhoms from different species (Fig. 1b).

First, we designed an iRhom2 construct without the IRHD (miR2_ΔIRHD), to study the function of the IRHD. We replaced the IRHD with a flexible linker to avoid a possible structural disruption by fusing TMH1 directly to TMH2. Additionally, we designed constructs which only lack one of the two structured regions (miR2_IRHD1 without IRHD2 and miR2_IRHD2 without IRHD1) to test whether the IRHD consists of two independent domains (Fig. 1b). The IRHD was divided according to the cysteine residue positions to ensure an even number of cysteine residues in both parts to ensure disulphide bond formation.

The main and best described function of iRhoms is their interaction with ADAM17, which is necessary for ADAM17 forward trafficking. Therefore, we firstly analysed our iRhom2 constructs with a focus on their ability to bind ADAM17 and promote its maturation. To study these constructs in vitro we first chose HEK293 cells expressing endogenous iRhoms. When expressing a murine wt_iRhom2 (wild-type) construct in HEK293 cells an increase of ADAM17 maturation was observed (Fig. 1c; Fig. S2a). Additionally, co-immunoprecipitations (co-IP) of wt_iRhom2 contained immature ADAM17 (imADAM17) and mature ADAM17 (mADAM17) (Fig. 1c; Fig. S2b). In contrast, deletion of the whole IRHD or of one of the two structured IRHD parts (miR2_ΔIRHD, miR2_IRHD1 and miR2_IRHD2) neither promoted ADAM17 maturation nor interacted with ADAM17 (Fig. 1c; Fig. S2a,b). In conclusion, the domain integrity of the IRHD seems to play an indispensable part in the binding between ADAM17 and iRhoms. However, at this stage we cannot rule out that the deletion/replacement of the whole IRHD or parts of it critically affects the structure of the rhomboid core and thereby the interaction interface between TMH1 and the ADAM17 TMH.

In order to investigate how correct folding of IRHD affects the entire iRhom protein and its main function, the ability to bind and regulate ADAM17, we wanted to deliberately compromise the structural integrity of the IRHD. To analyse the susceptibility of iRhom functions to changes in the structural integrity of the IRHD we focused on highly conserved parts within the structured parts of the IRHD, which were shown to be structured in our secondary structure prediction (Fig. 1b). A single point mutation was introduced into the middle of the first helix of the IRHD. Here, a conserved leucine residue (a classical helix builder) was replaced with a proline residue (miR2_L438P), a classical helix/structure breaker. This simple single point mutation was already sufficient to critically affect IRHD and especially the iRhom functions with respect to ADAM17, since this mutant neither binds ADAM17 nor promotes its maturation (Fig. 1c; Fig. S2a,b). Furthermore, while there was surface expression observed for wt_iRhom2, none was detected for miR2_L438P (Fig. 1d), which strongly hints towards a failure to leave the ER most probably due to ER quality control triggered by the misfolded IRHD. Accordingly, our different IRHD deletion mutants are also not expressed on the cell surface (Fig. S2c). This fact could indicate a structural problem that triggers the ER quality control or another effect that prevents the iRhom2 deletion mutants from reaching the cell surface such as mispositioning of the remaining parts caused by the deletions.

We concluded that the IRHD and especially its structural and domain integrity is indispensable for iRhom functions regarding binding to ADAM17 and promoting its maturation. A general overview of all mutants analysed in this study and their effects can be found in Fig. S1.

### Mutation in a highly conserved but unstructured motif of the IRHD has a dominant negative effect on ADAM17 maturation

**Fig. 2:**
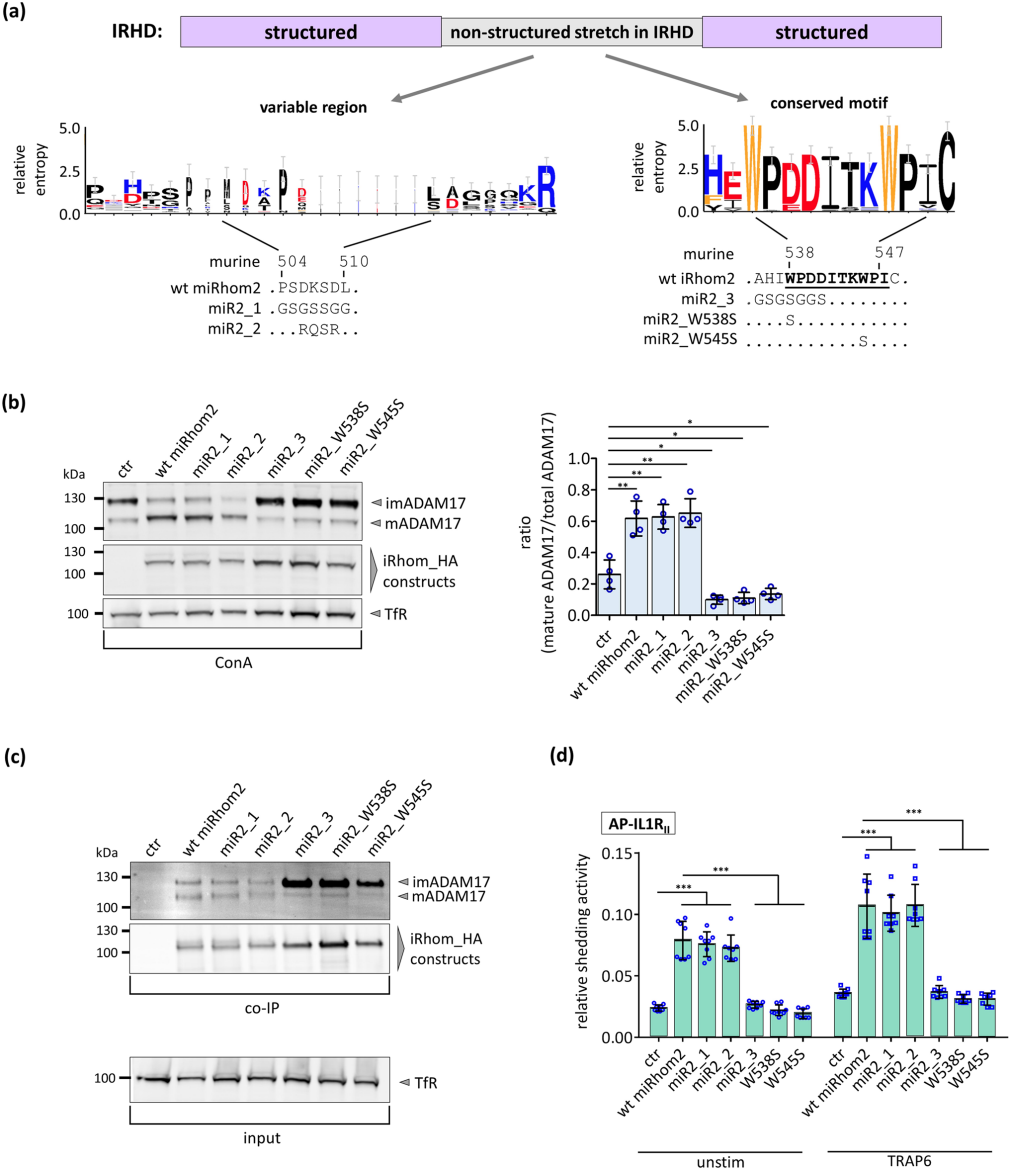
Impact of mutations within the IRHD on ADAM17. **a** The conserved motif and a highly variable sequence within the non-structured stretch are represented as weblogo presentation. Noteworthy, the fact that some amino acid residues such as tryptophan and cysteine residues usually appear in low frequency in protein sequences leads to a higher scoring when they are highly conserved. Designed mutations of the murine iRhom2 are shown. The construct miR2_1 and miR2_2 were designed by mutating the region located N-terminally from the conserved motif either by replacement with a flexible linker (miR2_1) or by changing charges of three amino acid residues (miR2_2) to create a drastic change at this position. To generate miR2_3 parts of the conserved motif were replaced with a flexible linker. **b**–**d** HEK293 cells stably expressing the indicated iRhom constructs or GFP as negative control (ctr) were used for the described experiments. The transferrin receptor (TfR) served as input control. **b** To analyse the ADAM17 maturation, glycosylated proteins were enriched via ConA beads. Maturation was assessed by densitometric measurements and calculation of the ratio between mADAM17 and total ADAM17 derived by the sum of mADAM17 and imADAM17. *n* = 4. **c** To analyse the binding between ADAM17 and iRhom constructs, co-IPs were performed by using the iRhom constructs (with HA tag) as bait. Quantitative analysis of ADAM17 binding can be found in figures S2d, e. *n* = 4. **d** ADAM17-mediated shedding activity was assessed by performing an alkaline phosphatase (AP) assay in HEK293 cells stably expressing the indicated iRhom construct. The ADAM17 substrate IL-1R_II_ tagged with AP was used. Since ADAM17 activity can be upregulated by stimulation of certain G-protein coupled receptors, HEK293 cells were stimulated with the PAR1 (protease activated receptor 1) agonist TRAP6. Cells were incubated for 2 h under unstimulated condition or stimulated with TRAP6. In all cell lines with expression of iRhom2 constructs, wt_iRhom2 or control there was a significant stimulation of ADAM17-mediated shedding by TRAP6 (*p* < 0.01). *n* = 8

Next, we focused on the non-structured stretch within the IRHD (Fig. 1b, grey), since such regions often act as regulatory hubs [34]. Here, most positions show a high evolutionary variability. However, there also exists a short, highly conserved motif within this non-structured stretch (Fig. 1b). To test whether this conserved motif plays a critical role for iRhom functions, we replaced parts of this motif with a flexible linker to preserve the unstructured characteristics (miR2_3) (Fig. 2a). As controls two additional iRhom2 constructs were designed (Fig. 2a), in which parts of the variable regions within the non-structured stretch in close proximity to the conserved motif were mutated (miR2_1 and miR2_2) (Fig. 2a, Fig. S1). By reversing the charge of the amino acids within this region miR2_2 has the most drastic mutation. Despite these mutations both controls behave exactly like wt_iRhom2 in terms of ADAM17 binding and promoting ADAM17 maturation compared to the wt_iRhom2 (Fig. 2a, c). The fact that iRhom functions are not susceptible for mutations within this part of the non-structured stretch is in line with its high evolutionary variability (Fig. 1b). Moreover, these results support our secondary structure analysis, since the predicted non-structured stretch has no effect on the folding of the IRHD.

In contrast, the construct in which the conserved motif within the non-structured stretch is mutated (miR2_3) lost the ability to promote ADAM17 maturation (Fig. 2b). Nonetheless, miR2_3 did not lose all functions. Instead, it had a dominant negative effect on ADAM17 maturation (Fig. 2b). This was indicated by the increase in imADAM17 and a decrease in mADAM17. Moreover, co-IPs demonstrated that miR2_3 still binds significantly more total ADAM17. This was due to increased binding of imADAM17 (Fig. 2c; Fig. S2d) while binding to mADAM17 is significantly decreased. (Fig. 2c; Fig. S2e).

Interestingly, the conserved motif possesses two highly conserved tryptophan residues, which usually appear in a low frequency in proteins and have a high evolutionary substitutability [35] (Fig. 2a). We replaced these residues with serine residues to cause a substantial change in the physicochemical properties. Both iRhom2 mutants (miR2_W538S, miR2_W545S) and a double mutant (miR2_W538S_W545S) showed the same effect on ADAM17 maturation and binding as miR2_3 (Fig. 2b, c; Figs. S2d,e, S3a,b,c). Additionally, mass spectrometry measurements revealed that mutation of the conserved motif does also not affect the binding to other iRhom interactors such as FRMD8 or 14-3-3 proteins [21–23] (Fig. S4a, b). Especially, the unaltered binding to FRMD8 is noteworthy, since it is a crucial stability factor for iRhoms. These results indicate that the conserved motif is not required for all iRhom functions in general but rather plays an isolated role for iRhom-mediated ADAM17 maturation.

To test whether the identified sequence is functionally conserved, we also introduced the same single point mutations into human iRhom2 (hiR2_W567S and hiR2_W574S). Expression of any of these constructs had the same effect as miR2_3 on maturation and binding of ADAM17 (Fig. S3d, e, f).

Overall, these results show that these mutations within the unstructured region do not affect the integrity and function of the entire iRhom2 protein. Instead, mutations within the evolutionary variable part appear to have no effect at all. Interestingly, mutations within the highly conserved part of the unstructured region specifically abolish the ability of iRhom2 to promote the maturation of ADAM17 without affecting other functions such as ADAM17 binding.

### Disruption of the highly conserved motif does not interfere with ADAM17-mediated shedding

Next, we analysed whether the mutations in the conserved motif influence the shedding of the known ADAM17 substrate IL1R_II_ [36]. In line with their effect on ADAM17 maturation, wt_iRhom2, miR2_1 and miR2_2 showed increased levels of constitutive ADAM17-mediated shedding as well as induced ADAM17-mediated shedding in response to stimulation with the PAR1 (protease activated receptor 1) agonist TRAP6 (Fig. 2d; Fig. S5a). In contrast, miR2_3, miR2_W538S and miR2_W545S did not show elevated ADAM17-mediated shedding compared to wt iRhom2 (Fig. 2d). However, in comparison to the control lacking any exogenous iRhoms, these mutations did not cause a further reduction in ADAM17 activity (Fig. 2d) despite the fact that a decrease in mature ADAM17 could be observed (Fig. 2b). This implies that the endogenous iRhoms and the remaining pool of mature ADAM17 are sufficient to maintain the basal shedding activity in cells expressing these mutants.

### iRhoms with disruption in the highly conserved motif do not rescue the absence of endogenous iRhoms

**Fig. 3:**
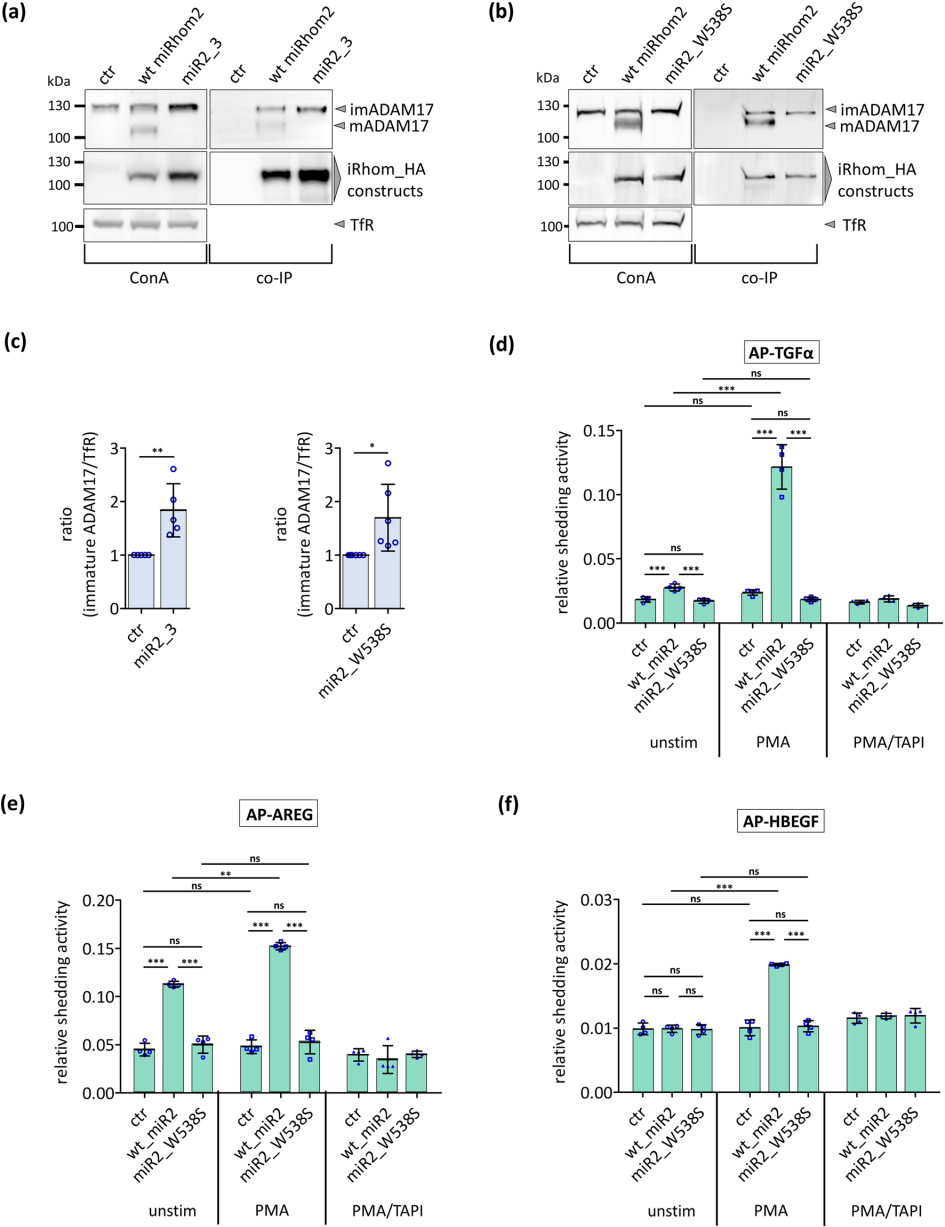
The highly conserved motif within the IRHD is crucial for ADAM17 maturation and activity in iRhom deficient cells. MEFs, deficient for iRhom1 and iRhom2, stably expressing the indicated iRhom constructs were used for the described experiments. As negative control, cells stably expressing GFP (ctr) were used. **a**–**b** To analyse the maturation of ADAM17, glycosylated proteins were enriched by concanavalin A beads (ConA) and immunoblotted. The transferrin receptor (TfR) served as input control. To analyse the binding between ADAM17 and iRhom constructs, co-IP were performed by using the iRhom constructs (with HA tag) as bait. Quantitative analysis of ADAM17 binding can be found in figures S5b, c. *n* > 3. **c** Relative amount of imADAM17 from conA precipitations was assessed by densitometric measurements from immunoblots (**a**) and (**b**). The ratio between imADAM17 and TfR was calculated and normalised to wt miRhom2. n > 5. **d**–**f** ADAM17-mediated shedding activity was assessed by an AP assay in iRhom1/iRhom2-deficient MEFs. The ADAM17 substrates transforming growth factor (TGFα) (**d**), amphiregulin (AREG) (**e**) and heparin-binding EGF-like growth factor (HBEGF) (**f**) each tagged with AP were utilised. Cells were incubated for 2 h under unstimulated condition, stimulated with the phorbol ester PMA (phorbol-12-myristate-13-acetate) or stimulated with PMA and treated with the inhibitor TAPI1. *n* = 4

To analyse further the influence of the conserved motif for iRhom function in an iRhom-deficient background, we expressed wt_iRhom2, miR2_3 or miR2_W538S in mouse embryonic fibroblasts (MEFs) derived from iRhom1/iRhom2-deficient mice [17]. While wt_iRhom2 rescued the maturation of ADAM17, neither miR2_3 (Fig. 3a) nor miR2_W538S did (Fig. 3b). In line with our previous results in HEK293 cells (Fig. 2b), the expression of both mutants (Fig. 3a, b, c) in these MEFs caused an enrichment of immature ADAM17. Again, both mutants still bound imADAM17 (Fig. 3a, b; Fig. S5b,c).

Next, we analysed ADAM17 mediated shedding of IL-1R_II_ in these iRhom1/iRhom2-deficient MEFs. Expression of wt_iRhom2 restored ADAM17 maturation and also shedding the ADAM17 substrate TGFα under unstimulated and stimulated conditions (Fig. 3d). In contrast, neither constitutive nor stimulated ADAM17-mediated TGFα shedding was observed in cells expressing miR2_W538S (Fig. 3d). Moreover, we also analysed other, physiologically relevant ADAM17 substrates such as different EGFR ligands and cytokine receptors such as IL6R. All these substrates were released from the cell surface when wt_iRhom2 was expressed (Fig. 3e, f; Fig. S5d). In contrast, miR2_W538S did not restore ADAM17-mediated shedding activity.

These results confirm that disruption of the conserved motif in the IRHD of iRhoms disturbs the maturation of ADAM17 but not the interaction between both proteins. The fact that the mutants still bind the ER-resident imADAM17 and that there is an enrichment of imADAM17 in the cells, seems to indicate that these proteins cannot be transported from the ER to the Golgi.

### The highly conserved motif in IRHD is indispensable for surface expression of iRhoms and ADAM17

**Fig. 4:**
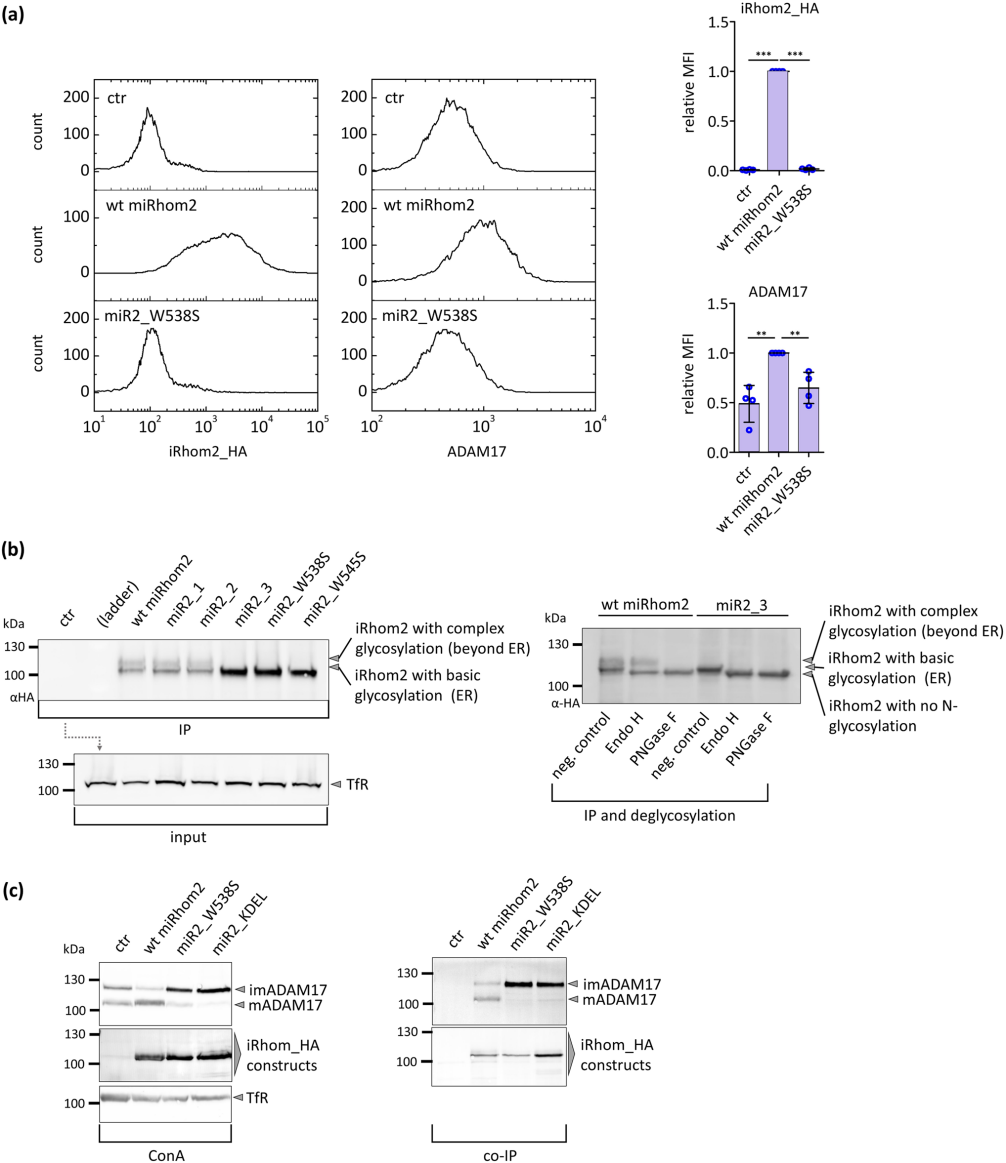
The integrity of the conserved motif within the IRHD is crucial for ER to Golgi transport. HEK293 cells stably expressing the indicated iRhom constructs or GFP (ctr) were utilised. **a** Cell surface expression of iRhom constructs and endogenous ADAM17 were measured by flow cytometry. For quantification, the geometric mean of the fluorescence intensity was normalised to the wt miRhom2 sample. *n* = 4. **b** Due to glycosylation murine iRhom2 is detectable as two separate bands by western blotting. Left panel: Different iRhom constructs were immunoprecipitated (IP) and immunoblotted. Right panel: Deglycosylation experiments were performed with immunoprecipitations of wt miRhom2 and miR2_3, and analysed by immunoblotting. IP samples were treated with either Endo H or PNGase F. As negative control samples were left untreated. The results show that the upper band of wt_iRhom2 is resistant to the treatment with endo H while the lower band slightly shifts to a lower molecular weight, which is the fraction without N-glycosylation. Treatment with PNGase F shifts the upper band also to the molecular weight of the fraction without N-glycosylation. *n* = 3. **c** To study the maturation of ADAM17, glycosylated proteins were enriched by concanavalin A beads (ConA). To specifically analyse the binding between ADAM17 and iRhom constructs, co-immunoprecipitations (co-IP) were performed by using the iRhom constructs (tagged with HA tag) as bait. Maturation and binding were assessed by immunoblotting. The transferrin receptor (TfR) served as input control. ADAM17 maturation was evaluated by comparing the amount of immature (imADAM17) and the amount of mature ADAM17 (mADAM17). Quantitative analysis of ADAM17 maturation levels and ADAM17 binding can be found in figures S7c, d. *n* > 3

Since we found that mutations in the conserved motif within the non-structured stretch (of the IRHD) abrogated ADAM17 maturation and activity, we analysed the cell surface expression of the iRhom constructs and endogenous ADAM17. While there was wt_iRhom2 construct observable at the cell surface, miR2_W538S was not detected (Fig. 4a). Furthermore, only wt_iRhom2 was able to enhance ADAM17 surface expression (Fig. 4a). Noteworthy, wt_iRhom2 or miR2_W538S did not alter the surface expression of proteins in general, since the surface expression of ADAM10, which is the evolutionary closest related protease to ADAM17, was not altered (Fig. S6a).

We also tested human iRhom2 and its corresponding mutants for their cell surface expression. Again, while the wt construct was present at the cell surface, the mutants were comparable to the negative control (Fig. S7a,b). These results are in line with our previous results and indicate that mutations within the conserved motif disrupt the ability of iRhoms to traffic through the secretory pathway and reach the cell surface.

### The highly conserved motif in IRHD is indispensable for the transport into the Golgi

To test whether the conserved motif is indispensable for transport into the Golgi, we took advantage of the circumstance that murine wt_iRhom2 expressed in HEK293 cells can be detected as two distinct bands via western blotting (Fig. 4b). We suspected that the upper band is a glycosylated form. Interestingly, while the iRhom variants with mutations in the variable region (miR2_1, miR2_2) also had this second band, it was absent from all mutants with a disrupted conserved motif (Fig. 4b). We utilised a glycosidase assay (Fig. 4b) to determine where in the secretory pathway the protein fraction of the upper band is located. Since ER-acquired N-glycosylation is sensitive to Endo H but not when it is further modified in the Golgi, the digestion with this glycosidase allows determining where in the secretory pathway a protein is located. In contrast, treatment with peptide PNGase F removes every N-glycosylation. Indeed, we found that the upper band represents protein modified in the Golgi, while the lower band possess only glycosylation acquired in the ER (Fig. 4b) indicating that the upper band represents the iRhom2 fraction, which is located in the Golgi or already passed it. In contrast, the only band present for miR2_3, miR2_W538S or miR2_W545S, respectively, showed only glycosylation acquired in the ER. Thus, for iRhom2 missing an intact conserved motif only the fraction glycosylated in the ER can be detected. This clearly demonstrates that these mutants do not reach the Golgi. This is also in line with the earlier demonstrated missing ADAM17 maturation (Fig. 2b, 3a, b), since the maturation also takes place in the Golgi.

To further verify whether mutations in the conserved motif impair the transport to the Golgi, we compared miR2_W538S with an iRhom2 construct with the ER-retention sequence KDEL (miR2_KDEL). As shown in Fig. 4c (and Fig. S7c) miR2_KDEL had the same effect on ADAM17 maturation as miR2_W538S. Moreover, both constructs showed the same interaction with ADAM17 (Fig. 4c; S7d). A density gradient experiment to separate Golgi from ER also showed that only wt murine iRhom2 was present in pure Golgi fractions, while miR2_W538S can only be found in ER containing fractions (Figure S7e).

Overall, these findings support the hypothesis that mutating the conserved motif in the IRHD impairs ER to Golgi transport. We, therefore, termed this motif iCERES (iRhom Conserved ER to Golgi Export Sequence).

### Mutating iCERES does not lead to misfolded IRHD and does not make iRhom a target for ER quality control

As we demonstrated above, mutations within iCERES do not abrogate interaction of iRhom with ADAM17 (Fig. 1c, d). This already indicates that mutations within iCERES do not lead to a globally misfolded IRHD. However, it might still be possible that these mutations cause misfolding in the IRHD which would be recognised by the ER quality control and trigger ERAD (ER-associated degradation).

One of the main steps of the ER quality control for N-glycosylated proteins destined to be transported through the secretory pathway is the binding to either calnexin or calreticulin, which are both lectin chaperones. Correctly folded proteins can leave the calnexin- and calreticulin-mediated quality control system and enter the ER exit sites (ERES) where COP vesicle-dependent anterograde transport to the Golgi takes place [37]. Misfolded proteins on the other hand remain longer in the lectin chaperone system and undergo several folding attempts. If these attempts are unsuccessful the misfolded proteins subsequently undergo ERAD (Figure S8a).

**Fig. 5:**
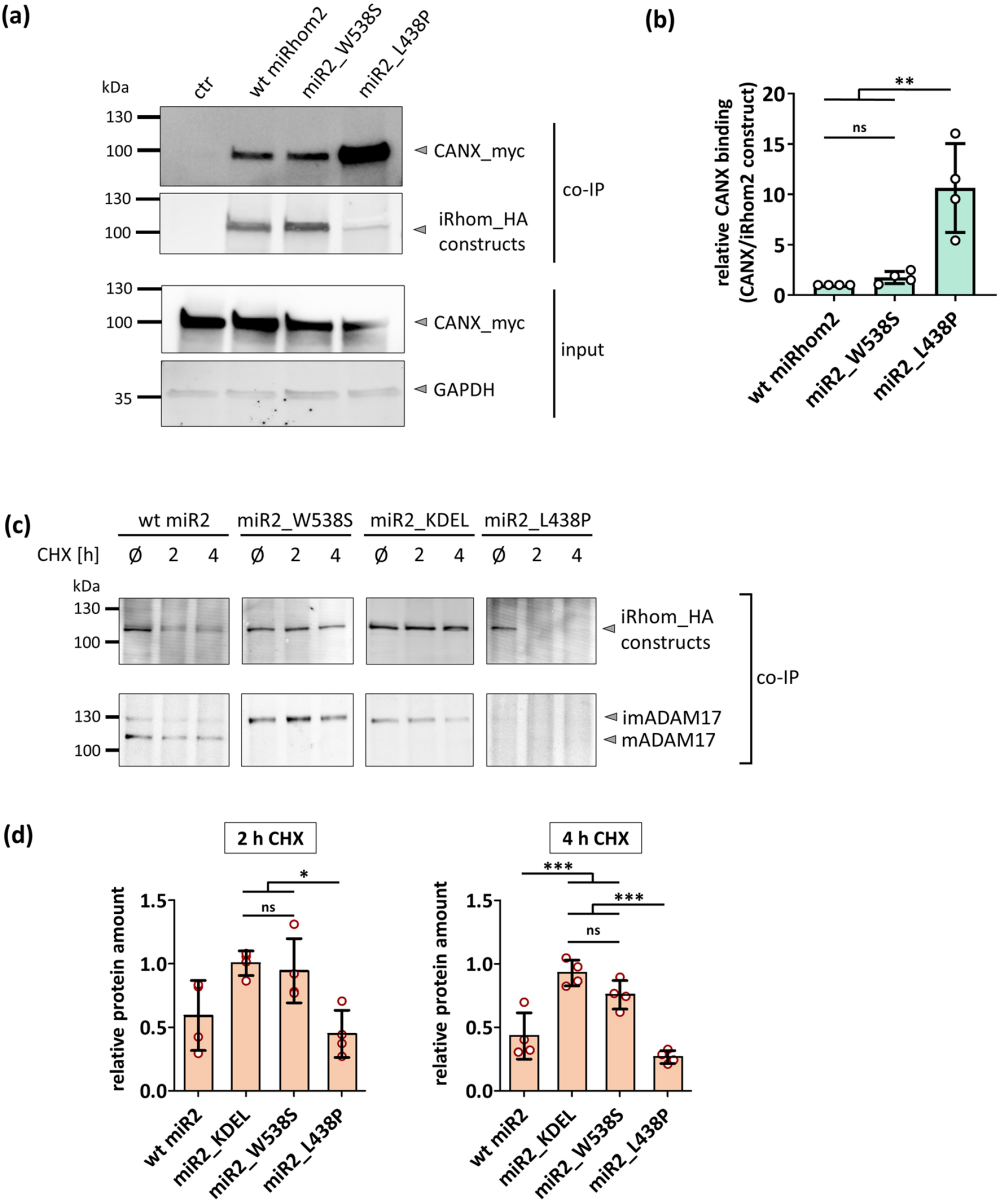
Mutations in iCERES do not cause misfolding of iRhom. **a** HEK293 cells stably expressing the indicated HA-tagged iRhom constructs were additionally transfected with myc-tagged calnexin**.** Co-IP experiments with the different iRhom2 constructs as bait were performed and analysed by western blotting using a α-myc antibody to probe for calnexin. **b** Immunoblot signals (**a**) were quantified and calnexin binding was determined by calculation of the signal ratio between calnexin and the respective iRhom2 construct. *n* = 4. **c**–**d** To analyse the half-life of the iCERES mutant miR2_W538S compared to wt miR2 and miR2_L438P, a cycloheximide-based pulse chase experiment was performed. *n* > 4. **c** Cells were lysed at the indicated time points after starting treatment with cycloheximide (CHX) and then analysed for iRhom2 constructs and ADAM17 by immunblotting. **d** For each construct **i**mmunoblot signals (**c**) were quantified and calculated in relation to the respective control without CHX at time point 0 h (Ø) which was set to 1

Since calnexin was found as an interactor candidate in our proteomic approach (Sup. Table 1), we tested whether we could confirm this interaction by co-IP and immunoblotting. Indeed, calnexin binds to wt iRhom2 (Fig. 5a). If mutations within iCERES cause misfolded IRHD, it would be expected that the iCERES mutant miR2_W538S is trapped longer in the calnexin-dependent ER quality system and hence shows more binding of calnexin. Strikingly, miR2_W538S binds calnexin in the same manner as wt iRhom2 (Fig. 5a,b), supporting that iCERES mutants are correctly folded. In contrast, the miR2_L438P construct, where the structured part of the IRHD was targeted, clearly shows significant more calnexin binding (Fig. 5a,b). We also tested our three iRhom2 deletion mutants miR2_ΔIRHD, miR2_IRHD1 and miR2_IRHD2 for calnexin binding. Since miR2_ΔIRHD lacks a luminal domain, calnexin should no longer bind. Thus, miR2_ΔIRHD can serve as a control that calnexin binding to iRhom2 constructs is specific to IRHD and its folding state. As expected, miR2_ΔIRHD shows no calnexin binding (Figure S8b). Interestingly, miR2_IRHD2 has the same calnexin binding as wt iRhom2 (Figure S8b). This suggests that the second part of the IRHD (without iCERES) can still achieve its correct structural fold. In contrast, miR2_IRHD1 shows significantly higher calnexin binding compared to wt iRhom2 (Figure S8b), which indicates folding problems when the second part of the IRHD is missing.

In conclusion, a point mutation in the structured part of the IRHD causes significant misfolding in iRhom2, which is recognised by ER quality control, whereas point mutations in iCERES do not.

### Mutating iCERES results in an increased half-life of iRhom2 and ADAM17

To further rule out that mutations in iCERES cause misfolding triggering ER quality control, we performed cycloheximide-based pulse-chase experiments. The clearance of misfolded proteins from the ER by ERAD is fast and efficient [37, 38], and pulse chase experiments of a wide variety of different transmembrane proteins, where mutations result in misfolding, revealed that already after one to two hours most of the unfolded protein is degraded [38–42]. As a control we used again the miR2_L438P mutation, which was deliberately designed to get misfolding and already showed more calnexin binding. As expected with a misfolded IRHD, which triggers ER quality control and ERAD, already 4 h after inhibition of biosynthesis by cycloheximide most of the misfolded miR2_L438P protein was degraded (Fig. 5c, d). As expected wt iRhom2 also shows a reduction in protein amount over time, since it can undergo its normal turnover: travelling to the surface, internalisation and recycling, as well as subsequently lysosomal degradation (Fig. 5c, d). In contrast, miR2_KDEL (wt iRhom2 with an additional KDEL motif) does not undergo normal turnover, since it is trapped in the ER. Since its IRHD is not mutated, it should not trigger ERAD. And indeed, it is not degraded and even forms still a stable complex with ADAM17 over the analysed time (Fig. 5c, d).

Next, we tested the iCERES mutant miR2_W538S and found that it is as stable as miR2_KDEL (with its intact IRHD) and is significantly different from the misfolded miR2_L438P (Fig. 5c, d). Furthermore, iCERES mutants form a stable complex with ADAM17 over the tested time and show also a stabilising effect on immature ADAM17 compared to wt iRHom2 (Fig. 5c, d). Moreover, miR2_W538S is also significantly more stable than wt iRhom2. This may be due to the lack of forward trafficking through the secretory pathway which prevents normal turnover.

To test whether immature ADAM17 bound to iCERES mutants is also stabilised, we performed an additional cycloheximide-based pulse-chase experiment with iRhom1/iRhom2-deficient MEFs to avoid interference with endogenous iRhoms. Again, we observed that the iCERES mutant is stable with a significantly increased half-life (Figure S9a,b). Furthermore, immature ADAM17 is significantly more stable in cells expressing an iCERES mutant compared to cells without any iRhom expression (Figure S9a,b). This is in line with our previous results, which showed a significant higher amount of immature ADAM17 in these MEFs expressing miR2_3 or miR2_W538S compared to the negative control (Fig. 3a–c). These results are also in line with our findings that miR2_W538S still interacts with FRMD8 (Fig. 4a, b), which is a crucial stability factor for iRhoms [22].

To further rule out the possibility that there could be a fraction of miR2_W538S molecules leaving the ER but undergoing accelerated lysosomal degradation due to possible instabilities introduced by mutations within iCERES, we used bafilomycin A1 to inhibit lysosomal degradation. The fraction of wt iRhom2 (with Golgi-acquired glycosylation) that left the ER and entered the secretory pathway was significantly enriched by bafilomycin A1 (Fig. S9c, d). As expected, inhibition of the lysosome leads to an accumulation of wt iRhom2 that has already left the ER. In contrast, under the same conditions, no miR2_W538S with Golgi-acquired glycosylation was detected with or without lysosomal inhibition (Fig. S9c, d). Furthermore, we performed the same lysosomal inhibition experiment in MEFs deficient for iRhom1 and iRhom2. Here, mature ADAM17 is significantly enriched, if the lysosomal degradation is blocked (Fig. S9e, f) in cells expressing wt miRhom2. In contrast, no mature ADAM17 can be detected in cells expressing miR2_W538S or a vector control (Fig. S9e, f). These lysosomal inhibition experiments again indicate that miR2_W538S molecules seem not to enter the Golgi and the secretory pathway and also do not promote ADAM17 maturation.

Overall, these data again clearly demonstrate that mutations within iCERES do not cause a misfolded IRHD which would be recognised by ER quality control. Furthermore, iCERES mutants have a longer half-life than wt iRhom2 and also stabilise ADAM17.

### *Mice with iRhom2*^*W538S/W538S*^* show impaired iRhom2-dependent ADAM17 activity*

**Fig. 6:**
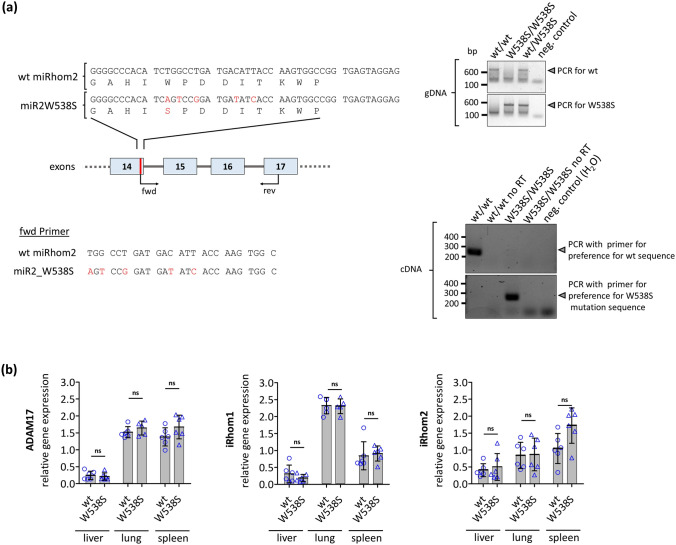
Generation of iRhom2^W538S/W538S^ mice. **a** The W538S mutation was inserted into exon 14 of the murine iRhom2 gene (RHBDF2) by CRISPR/Cas9. Genomic DNA (gDNA) and cDNA derived from generated mutant mice were analysed by PCR with primers specific for the wt or the W538S sequence. The possibility that introduced mutations accidentally altered the iRhom2 mRNA splicing was excluded by the fact that analysed cDNA of wt and W538S produced PCR products of the same size. As a negative control, H_2_O was used instead of mRNA. To exclude primer binding to residual genomic DNA, a second negative control was also used. Here, the reverse transcriptase (RT) was omitted from the reaction. **b** mRNA expression of the ADAM17, iRhom1 and iRhom2 in liver, lung and spleen from mutant mice was measured by qPCR. *n* = 6

To test the physiological relevance of iCERES, we generated mice with the W538S mutation in iRhom2 (Fig. 6a). The possibility that our mutation caused altered splicing was excluded by PCR analysis of transcripts (Fig. 6a). Additionally, a mouse line (Del) with a short deletion resulting in an early stop codon was generated and used as additional iRhom2 deficient control. All mouse lines were healthy, like reported earlier for iRhom2 deficient mice [15, 16]. Moreover, in both mouse lines the mRNA expression of ADAM17, iRhom1 and the respective iRhom2 variant were unchanged (Fig. 6b; Fig. S10a).

**Fig. 7:**
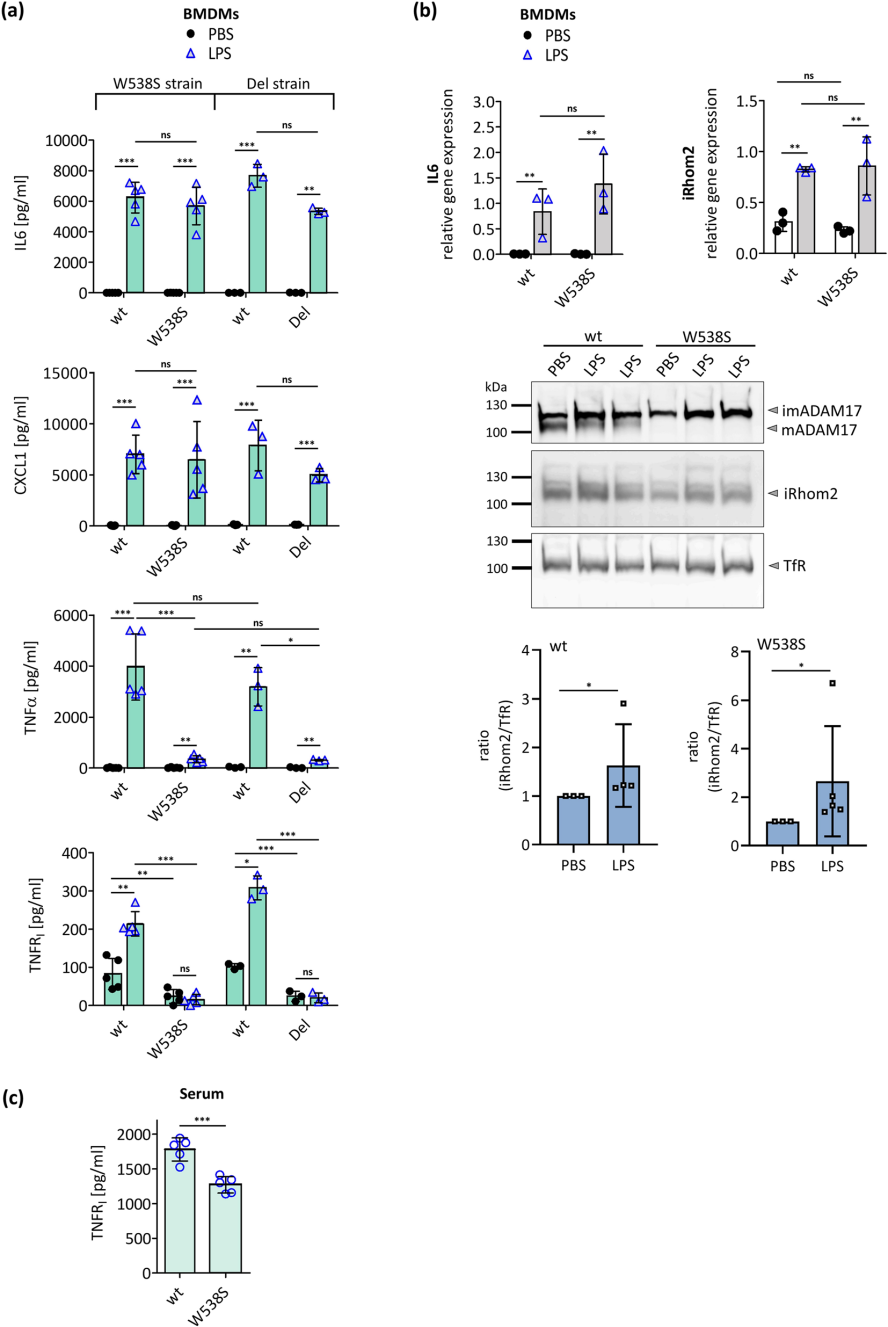
iRhom2-dependent ADAM17 activity in iRhom2^W538S/W538S^ mice. BMDMs from mice homozygous for either iRhom2_W538S or for an iRhom knock out (Del) and BMDMs from their respective wild type littermates (wt) were treated for 24 h with 100 ng/ml LPS or with PBS as negative control. **a** LPS-stimulated release of IL6, CXCL1, TNFα and TNFR_I_ were measured by ELISA. W538S: *n* = 5, Del: *n* = 3 **b** Induction of the IL6 and iRhom2 mRNA expression upon stimulation with LPS was measured by qPCR. *n* = 3. Additionally, upregulation of iRhom2 on protein level was determined. Expression of endogenous iRhom2 and ADAM17 in BMDMs was detected by precipitating glycosylated proteins with concanavalin A beads and immunoblotting. Transferrin receptor (TfR) served as input control. BMDMs were derived from mice homozygous for either iRhom2_W538S or wild-type (wt). *n* > 3. **c** TNFR_I_ levels in serum from iRhom2^W538S/W538S^ mice as well as from their wild type littermates were determined by ELISA. W538S: *n* = 5

Next, we isolated BMDMs (bone marrow-derived macrophages), in which iRhom2 is the dominantly expressed iRhom [15]. The release of neither IL6 nor of CXCL1 was altered in BMDMs^W538S/W538S^ or BMDMs^Del/Del^ after stimulation with the proinflammatory stimulus LPS compared to BMDMs^wt/wt^ (Fig. 7a). Additionally, the induction of IL6 mRNA expression by LPS remained unchanged (Fig. 7b). Hence, ADAM17-independent pathways do not seem to be affected. In contrast, release of soluble TNFα and soluble TNFR_I_ through ADAM17-mediated shedding was abolished in BMDMs^W538S/W538S^ and BMDMs^Del/Del^ (Fig. 7a). There was also an induction of iRhom2 mRNA expression upon stimulation with LPS in BMDMs^W538S/W538S^ and BMDMs^wt/wt^ (Fig. 7b).

On the protein level iRhom2 was clearly detectable in BMDMs^W538S/W538S^ but not in BMDMs^Del/Del^ (Fig. 7b; Fig. S10b). As expected, no mature ADAM17 was detectable in BMDMs^W538S/W538S^ or BMDMs^Del/Del^ (Fig. 7b; Fig. S10b). This is in line with our in vitro results and earlier findings from iRhom2 deficient mice [15]. Moreover, the protein levels of iRhom2 and ADAM17 in BMDMs^wt/wt^ and BMDMs^W538S/W538S^ increase after LPS stimulation in a manner consistent with the increased mRNA expression (Fig. 7b).

Additionally, we analysed serum for soluble TNFR_I_, which was reduced when ADAM17 activity is impaired in vivo [43]. We found a significant reduction in soluble TNFR_I_ in iRhom^W538S/W538S^ mice (Fig. 7c).

Overall, these results clearly show that a single-point mutation within iCERES of iRhom2 can abrogate ADAM17 activity not only in vitro but also in a physiological setting.

### iRhoms interact with proteins involved in vesicle transport

**Fig. 8:**
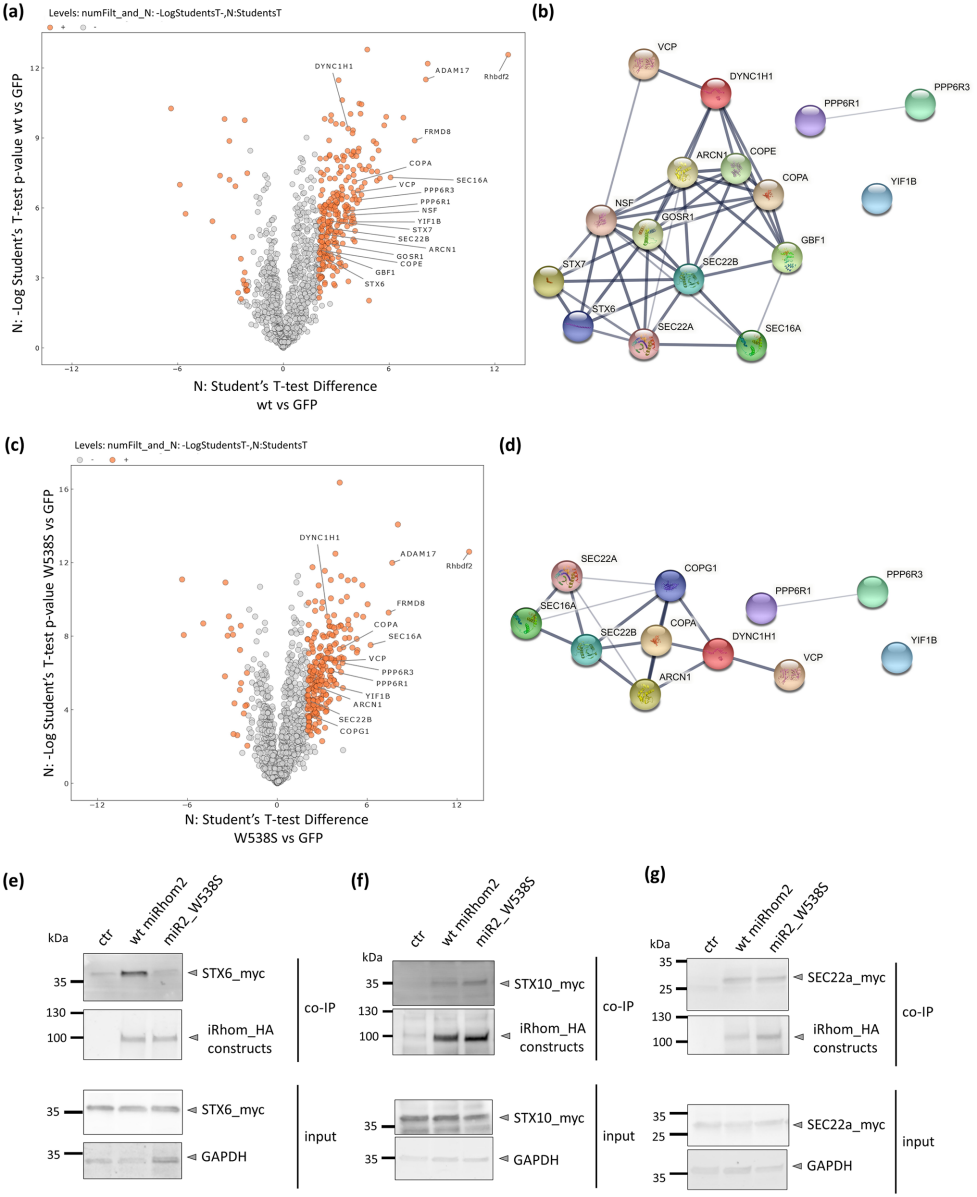
iRhom2 interacts with proteins involved in vesicle-mediated intracellular transport. Volcano plots of the quantitative comparison of **a** wild-type iRhom2 vs vector GFP (control) and **c** mutant iRhom2 (W538S) vs vector control (GFP) co-immunoprecipitations from HEK293 cells based on label-free quantification. Significant regulated proteins are labelled orange (requirements: *p*-value < 0.01, difference/ratio: > fourfold). All proteins belonging to the GO Group “endoplasmic reticulum to Golgi vesicle-mediated transport” (GO:0006888) and syntaxin 6 (STX6) and syntaxin 7 (STX) as well as the already known main iRhom2 interactors are labelled. The volcano plots were generated using Instant Clue [60]. **b**, **d** String images of all proteins belonging to the GO Group “endoplasmic reticulum to Golgi vesicle-mediated transport” (GO:0006888) as well as syntaxin 6 (STX6) and syntaxin 7 (STX7) are shown. All these proteins were found in the quantitative comparison of **b** wild type iRhom2 vs vector control (GFP) and **d** mutant iRhom2 (W538S) vs vector control (GFP) co-immunoprecipitations based on label-free quantification. (Requirements: *p*-value < 0.01, difference/ratio: > fourfold). Of note, SEC22A was not found in the interactome screen but identified by western blot (**f**). The images were obtained from String v11.0 (string-db.org) [61]. **e**–**g** HEK293 cells stably expressing the indicated HA-tagged iRhom constructs were additionally transfected with myc-tagged syntaxin 6 (STX6_myc) (**e**), syntaxin 10 (STX10_myc) (**f**) or SEC22a_myc (**g**). Co-IP experiments with the different iRhom2 constructs as bait were performed and used for western blotting. To probe for the myc-tagged proteins a α-myc antibody was used. Quantitative analysis binding to iRhom2 can be found in figures S10a, b, c. *n* = 3

We next analysed our interactome data derived from co-IPs from wt iRhom2 and miR2_W538S for interaction partners involved in ER to Golgi transport. We, therefore, focused our analysis of our proteomic interactome data (Sup. Table 1) on proteins belonging to the GO (gene ontology) group “endoplasmic reticulum to Golgi vesicle-mediated transport” (GO:0006888) (Sup. Table 2). Indeed, our proteomic approach revealed interaction with different proteins involved in vesicle formation and forward trafficking through the secretory pathway. We found a significant enrichment of proteins involved in ER to Golgi transport for wt iRhom2 as well as miR2_W538S (Fig. 8a–d). Strikingly, there is a significant enrichment of SEC16a, which is a crucial part of ERES (ER exit sites) and COP-II vesicle forward trafficking [44], in wt iRhom2 and in miR2_W538S samples. Furthermore, we found for both iRhoms additional shared interactors, which also play a crucial role in the ER-Golgi intermediate compartment (ERGIC), such as the coatomer subunit COPA and SEC22b. Overall, this means both, wild-type and iCERES mutants, seem to be able to enter the ER exit sites and iCERES mutants may even enter ERGIC like wt iRhom2. While wt iRhom2 with its cargo ADAM17 is then released into the Golgi and can further follow the secretory path as shown in our previous experiments, iCERES mutants seem to be held back, possibly because a gatekeeper factor is missing, which interacts with an intact iCERES. Most likely, this luminal factor is only transiently needed for Golgi entry and is not easily detectable with the available reagents.

Exclusively for wt iRhom2 we found NSF, GBF1 and GOSR1 as interactors (Fig. 8a–d), which are also involved in the ER to Golgi forward trafficking. Since these proteins have no luminal part it is not possible that the IRHD directly interacts with these proteins. Hence, it is unlikely that these proteins are responsible for the observed iCERES phenotype. Since these proteins are also implicated in intra-Golgi trafficking, it is more likely that they bind iRhoms in the Golgi and hence cannot reach iCERES mutants.

However, it is an important observation that members of the wide family of the soluble NSF attachment protein receptors (SNARE) seem to interact with iRhoms (Fig. 8a–d). SNARE proteins play a key role in intracellular vesicle transport, more precisely in vesicle fusion by physically bringing the membranes sufficiently close to fuse [45]. In our proteomic data we, for instance, found SEC22b, syntaxin 6 and syntaxin 7 (Fig. 8a–d, Sup. Table 1). Furthermore, we found the SEC22b homolog SEC22a and could also confirm syntaxin 6 interaction via additional co-IP experiments (Fig. 8e,g; Fig. S10c, d). Interestingly, syntaxin 6 as a well-established trans-Golgi marker interacts only with wt iRhom2, but not with the iCERES mutant miR2_W538S. Most likely this is due to the Golgi localisation of syntaxin 6. Most likely, syntaxin 6 may be involved in transferring iRhom2 and its cargo ADAM17 from the Golgi to the cell surface. This finding can be regarded as another supporting evidence that iCERES mutants cannot efficiently enter the Golgi.

In contrast to syntaxin 6, the ER- and ERGIC-localised SEC22a and SEC22b still bind to miR2_W538S. While the function of SEC22a is not yet understood the other members of the SEC22 family are crucially involved in ER to Golgi trafficking [46]. Moreover, we found with additional co-IP experiments that syntaxin 10 binds wt iRhom2 as well as miR2_W538S (Fig. 8f; Fig. S10e). While syntaxin 10 is described to be mainly localised in the retrograde transport, especially between endosomes and Golgi [47], it was also reported that it can be found in the ERGIC [48]. It seems that iRhoms already interact with syntaxin 10 between ER and Golgi. (Fig. 8f).

Overall, our results demonstrate that iRhoms interact with different proteins involved in vesicle transport, especially SNARES, when travelling through the secretory pathway. Furthermore, iCERES mutants can still enter ERES and even reach the ERGIC while still interacting with different transport-associated proteins. However, disruption of iCERES prevents iRhom from entering the Golgi.

## Discussion

The pseudoprotease iRhom is indispensable for ADAM17 maturation and thereby plays critical regulatory functions in inflammatory and proliferative diseases that depend on ADAM17-mediated shedding of cytokines, growth factors and their receptors. Using in silico and in vitro analysis of human and murine iRhom2 constructs we analysed the luminal iRhom homology domain (IRHD) and could show that this domain and its structural integrity is crucial for ADAM17 binding. This is in line with a previous report that already indicated the IRHD as important for iRhom functions [21]. While, as reported before, the TMH1 of iRhoms seems to provide the binding interface for efficient ADAM17 binding [19], the IRHD may provide the scaffold for a correct positioning of the TMH1 and/or an additional binding interface. Yet, a correctly folded IRHD is also indispensable for a stable iRhom protein. A structure-disrupting single-point mutation within the first helix of the IRHD prevents the production of a correctly folded and functional iRhom. Hence, this mutant cannot pass the ER quality control and is rapidly degraded. Interestingly, the deletion mutants of iRhom2 provide further insight into IRHD. Deletion of the entire IRHD (miR2_ΔIRHD) resulted in a loss of surface expression, probably due to the absence of iCERES. In addition, ADAM17 binding was abolished, again supporting the presence of important determinants for ADAM17 interaction within the IRHD. The deletion mutant miR2_IRHD2, which lacks the first part of the IRHD (including iCERES), still appears to be correctly folded, suggesting that this part is at least structurally an independent entity. Since iCERES is missing here, it is not surprising that this deletion shows no surface expression. Furthermore, miR2_IRHD2 does not bind to ADAM17, suggesting that the first part of the IRHD or the presence of both IRHD parts is crucial for interaction with ADAM17. Interestingly, miR2_IRHD1 (lacking the second part of the IRHD) appears to have folding problems within the ER, resulting in increased calnexin binding, abrogated surface expression and lost ADAM17 binding. It seems clear that both parts of the IRHD are necessary for a fully functional iRhom2. However, deletions in general remain only crude approaches. As it can neither be determined how the positioning of the other IRHD parts is affected by the chosen deletions nor how this in turn may affect iRhom functions, further characterisation of the structured and unstructured parts of the IRHD is required.

**Fig. 9:**
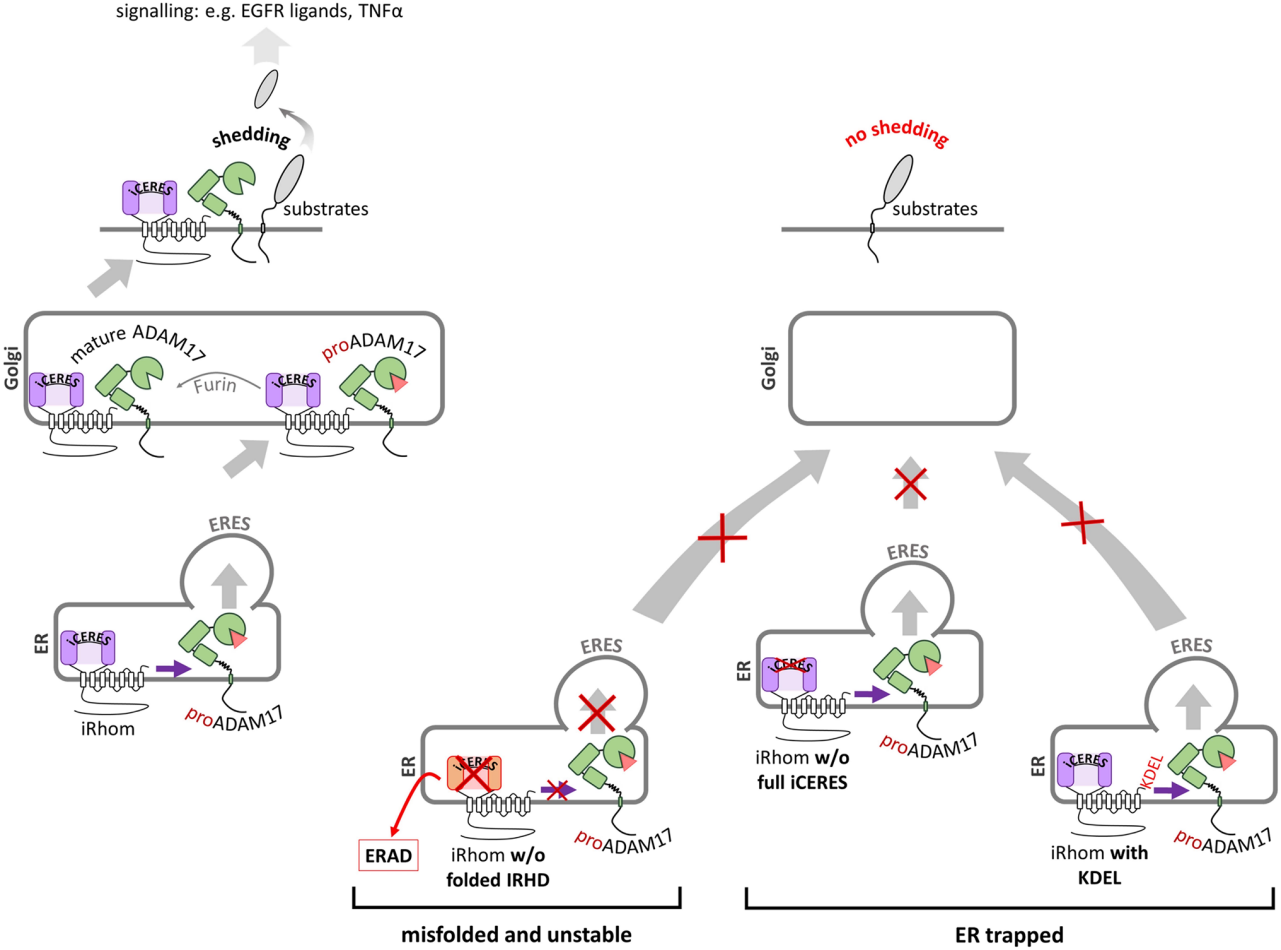
The IRHD and the conserved motif iCERES are indispensable for ADAM17 maturation and activity. Wild type iRhoms bind immature ADAM17 in the ER and shuttle it to the Golgi by COP-II vesicle-dependent anterograde transport. In the Golgi ADAM17 maturation takes place. Afterwards, mature ADAM17 can reach the cell surface and can shed its substrates enabling a wide variety of different signalling pathways such as the TNFα and the EGFR pathways. When the structural integrity of the IRHD is compromised, the whole iRhom protein is functionally impaired regarding ADAM17 binding and transport. Moreover, misfolded iRhom is recognised by ER quality control and cannot enter ER exit sites (ERES). Instead, it undergoes rapid ERAD. In contrast, iRhoms tagged with the ER retention signal KDEL and iRhoms with disrupted iCERES can still bind immature ADAM17. They are not recognised by ER quality control and still enter ERES but cannot be transported into the Golgi

By further analysing the IRHD, we were able to pin down highly conserved motif within a non-structured part of the IRHD which seems to be important for ER to Golgi transport (Fig. 9), which we termed iCERES (iRhom conserved ER to Golgi export sequence). We showed that the two tryptophan residues in iCERES are essential for its function. In this study we provided multiple pieces of evidence that iCERES is indispensable for ER to Golgi transport and hence for ADAM17 activity. Single point mutations targeting at least one of the tryptophan residues specifically abrogate ER to Golgi transport of iRhom2. We supported this by showing missing Golgi-dependent glycosylation, loss of interaction with a Golgi-located binding partner and loss of cell surface expression. In close association, the disruption of ER to Golgi forward trafficking by mutating iCERES in iRhom2 was mirrored by a loss of ADAM17 forward trafficking, maturation, cell surface expression and shedding activity. Strikingly, when iCERES is disrupted, the interaction with ADAM17 remains unaffected. This leads to entrapment of immature ADAM17 with iCERES-impaired iRhoms before the Golgi. Hence, iCERES-impaired iRhoms exert a dominant-negative effect on maturation, by not allowing endogenous iRhoms to access immature ADAM17 and promote its maturation.

Furthermore, we showed using different methods that a mutation within iCERES does not lead to misfolding or destabilising of the IRHD or the whole iRhom protein, and does not trigger ERAD ruling out simple ER retention via ER quality control.

Simple misfolding of the IRHD of iRhom2 would of course also result in an abrogated ER to Golgi transport. Yet, this would be in stark contrast to the impact of the miR2_L438P mutation on ADAM17 binding, where the IRHD structure was intentionally targeted. iCERES mutants are still able to bind ADAM17 and other interactors. Since one main binding interface between ADAM17 and iRhom2 is the first iRhom TMH, it could be argued that this interaction might not be compromised when the IRHD is misfolded. However, if the ER quality control is triggered by IRHD misfolding and, therefore, responsible for the so far found phenotype of iCERES mutations, it would inevitably involve ERAD (ER-associated degradation).

Instead, we found that iCERES mutants show a significantly increased half-life and have a stabilising effect on ADAM17. Furthermore, an iRhom2 construct C-terminally fused with the ER retention signal KDEL behaved the same way like iRhoms with disrupted iCERES, further supporting our model that iCERES is crucial for the ER to Golgi transport (Fig. 9).

Overall, inactivation of iCERES, which, as we showed, is localised in a non-structured part of the IRHD, has only a local effect and has no overarching consequences on the structural integrity of the IRHD or the whole iRhom protein. In contrast, targeting a structural part of the IRHD with the L438P mutation clearly disrupts the IRHD structure and leads to misfolding and thus to a loss of iRhom functions and consequently to rapid degradation by ERAD. The miR2_L438P mutant is hence a prime example of what a misfolded iRhom would behave, which is in stark contrast to all mutations targeting iCERES or the surrounding unstructured region. This clearly supports the notion that iCERES is a standalone motif in iRhoms crucial for ER to Golgi transport.

We furthermore were able to validate the relevance of iCERES in a physiological setting by producing a mouse line with the W538S mutation of iRhom2. While we studied overexpression of mutated iRhom2 in our in vitro experiments, the iRhom2_W538S mice allowed us to study iRhom2 without functional iCERES at a physiological expression level. These mice seemed healthy at first glance, but their immune system must be critically impaired since their ability to release soluble TNFα in an ADAM17-mediated manner is abrogated. This was also true for iRhom2 knockout mice in the present study and in line with iRhom2 knockout mice previously described by others. These mice were found to be resistant against LPS-induced and TNFα-mediated septic shock [15, 16]. Furthermore, similar to hypomorphic ADAM17^ex/ex^ mice [43] our iRhom2^W538S/W538S^ mice have a lower serum level of soluble TNFR_I_. Since TNFα is crucial in chronic immune responses such as rheumatoid arthritis and psoriasis, it seems likely that the W538S mutation has some protective impact on disease development by abrogating release of proinflammatory soluble TNFα which may be in part influenced by also preventing anti-inflammatory effects of soluble TNFR_I_. This would be in line with the finding that iRhom2 deficient mice are protected from inflammatory arthritis [49]. Moreover, we demonstrated that ADAM17-mediated shedding of ligands of the EGF receptors is also abrogated when iCERES is compromised, further proving the importance of iCERES for several crucial signalling pathways.

It is noteworthy that other rhomboids only carry a short loop region (L1) with a length of about 30 amino acid residues instead of an IRHD, which only exists in iRhoms and has a length of about 250 amino acid residues. In active rhomboids, the L1 loop appears to be crucial for protease function. Here, deletion of the loop leads to abrogation of protease activity [50, 51]. Interestingly, the L1 loop is reported to be a crucial substrate recognition site [52–54]. Since we and others [21] have shown that the IRHD is also crucial for iRhom2 to interact with its main client ADAM17, the interaction function of this L1 position may be evolutionarily conserved. Notably, a tryptophan residue within a short W/xR sequence is highly conserved in the L1 loop of many other rhomboids. Although iCERES contains two crucial tryptophan residues, it is not yet clear whether there is an evolutionary link to the W/xR motif in other rhomboids. The L1 loop and the W/xR motif are partially immersed in the membrane and appear to play a critical role in protease activity [50, 55], while iCERES seems to play a crucial role in the forward trafficking of iRhom2 but not in ADAM17 interaction.

Overall, our findings add new puzzle pieces to the iRhom-ADAM17 axis and enforce the notion that different structural units of iRhoms have distinct functions. Previous studies showed that the cytosolic N-terminus contributes to stability of iRhom2 and regulation of ADAM17 activity on the cell surface and the TMH1 of iRhom2 is a crucial interaction interface for ADAM17 [19–23]. Our findings show that iRhom2 requires a structurally intact IRHD for ADAM17 binding and a complete iCERES within the IRHD for ER to Golgi transport. Moreover, the physiological relevance of iCERES is underscored by the results from the iRhom2^W538S/W538S^ mice. Our results so far show that the IRHD holds at least two functions: It harbours iCERES and it provides overall structural stability for iRhom.

Additionally, we found novel iRhom interaction partners, which are involved in forward trafficking through the secretory pathway. Here, especially the proteins from the SNARE family are of interest, which are involved in vesicle fusion. We found that iRhom2 interacts with different proteins involved in vesicle transport, especially SNAREs, at different subcellular location: For instance, in both the ER and ERGIC iRhoms interact with members of the SEC22 family, while in the Golgi they interact with Syntaxin 6. Since iRhoms represent a crucial ER-exit and forward trafficking receptor for ADAM17, the binding of iRhoms to SNARE proteins seems to represent a likely candidate mechanism for the forward trafficking of the iRhom-ADAM17 complex, which should be analysed in more detail. Yet, iRhoms rely on a regulatory mechanism facilitated by iCERES for ER to Golgi transport. iRhoms with a disrupted iCERES may be able to enter ERES and interact with COP-vesicle subunits and may even be transported to the ERGIC. Our findings that interaction with syntaxin 6, a well-established trans-Golgi marker, is compromised in iCERES mutants further supports our findings that the motif may in fact be required for transition into the Golgi. Interestingly, syntaxin 10, which is reported not only to be mainly localised in the retrograde transport between endosomal vesicles and Golgi but can also be found in the ERGIC [47, 48], still binds to iCERES mutants. This indicates that iRhom2 and syntaxin 10 seem to meet at an early stage between ER and Golgi.

Our findings represent the first report of factors involved in the forward trafficking of the iRhom-ADAM17 complex. While we found that iRhom2 as cargo receptor for ADAM17 differentially interacts with several proteins associated with intracellular vesicle transport throughout the secretory pathway, further investigations are needed to determine the regulatory mechanisms involved. Moreover, the highly conserved motif crucial for ER to Golgi transport has to be also analysed further. While cytosolic sequences in transmembrane proteins necessary for forward trafficking are well understood, luminal sequences are less well understood. Recently, the importance of the first three N-terminal amino-acid residues in soluble, luminal proteins were described to have an effect on ER export efficiency [56]. Moreover, some transmembrane proteins are known to also possess a motif crucial for ER export. Here the motif represents a C-mannosylation site. This not yet understood posttranslational modification occurs at the indole C2 carbon atom of a tryptophan residue (Furmanek and Hofsteenge, 2000; Shcherbakova et al., 2017). It was shown that C-mannosylation is a critical factor for ER exit and secretion of certain proteins such as proteins containing thrombospondin type 1 repeats. [57, 58]. Interestingly, this not fully understood motif also relies on tryptophan residues like iCERES in iRhoms.

iRhoms and their ability to leave the ER enable ADAM17-mediated shedding which is a crucial hub in development, regeneration and especially immunity. This explains why iCERES is evolutionarily highly conserved. Inflammation, while being a protective mechanism against infections, can have a negative impact on the quality of life and can be even life-threatening if dysregulated. Pathologies associated with inflammation can be acute such as sepsis or chronic diseases such as rheumatoid arthritis, inflammatory bowel disease and psoriasis. Importantly, the prevalence of these pathologies is predicted to rise further [59]. TNFα is one of the main therapeutic targets for treating chronic inflammation and antibody-based approaches to block TNFα are well-established but expensive treatments. So far, the effort to directly inhibit the release of TNFα by using small-molecule inhibitors failed due to lacking specificity. Here, directly blocking ADAM17- iRhom2 binding and/or ADAM17 transport through the secretory pathway by targeting the IRHD and/or iCERES of iRhom2 in immune cells might be a promising alternative. Targeting iRhom2 may also be useful for finding treatments for other iRhom2-related pathologies such as tylosis with esophageal cancer (TOC). Therefore, it is essential to understand the underlying mechanism of iCERES in more detail to develop putative therapeutic strategies.

## Supplementary Information

Below is the link to the electronic supplementary material.

Supplementary file 1: Figure S1: Overview of all mutants used in this study. The relative position in the IRHD is schematically shown for all used mutants. Additionally, the effects of the different mutants on iRhoms are listed. Deletions are labelled yellow, single-point mutations are labelled red, and replacements of more than one amino acid residue are labelled blue. (TIF 2529 KB)

Supplementary file 2: Figure S2: HEK293 cells stably expressing the indicated iRhom constructs were utilised. As negative control, cells stably expressing GFP (ctr) were studied in parallel. (a) Maturation of ADAM17 depicted in figure 1c was assessed by densitometric measurements and calculation of the ratio between mADAM17 and total ADAM17 composed of mADAM17 and imADAM17. n = 3. (b) Binding of ADAM17 to the indicated iRhom constructs shown in figure 1c was assessed by densitometric measurements and calculation of the ratio between total ADAM17 and the respective iRhom construct. n = 3. (c) Cell surface expression of indicated iRhom constructs was measured by flow cytometry. For quantification, the geometric mean of the specific fluorescence signal was determined and normalised to that of wt iRhom2. n = 3. (d), (e) Binding of ADAM17 to the indicated iRhom constructs depicted in figure 2c was assessed by densitometric measurements and calculation of the ratio between total ADAM17 (d) or mADAM17 (e) and the respective iRhom construct. n = 4. (TIF 1745 KB)

Supplementary file 3: Figure S3: HEK293 cells stably expressing the indicated iRhom constructs were utilised. As negative control, cells stably expressing GFP (ctr) were used. (a), (d) To analyse the maturation of ADAM17, glycosylated proteins were enriched by using concanavalin A beads (ConA). To analyse the binding between ADAM17 and iRhom constructs, co-immunoprecipitations (co-IP) were performed by using the iRhom constructs (tagged with HA tag) as bait. Maturation and binding were assessed by immunoblotting. The transferrin receptor (TfR) served as input control. For (d) the western blots were cropped to exclude lanes, which are not subject of this manuscript. The uncropped version of these western blots can be found in the supplement file with all raw, uncropped western blots of this manuscript. (b), (e) Maturation of ADAM17 in (a) and (d) was assessed by densitometric measurements and calculation of the ratio between mADAM17 and total ADAM17 composed of mADAM17 and imADAM17. n > 3. (c), (g) Binding of ADAM17 to the indicated iRhom constructs in (a) and (d) was assessed by densitometric measurements and calculation of the ratio between mADAM17 and the respective iRhom construct. n = 3. (TIF 2228 KB)

Supplementary file 4: Figure S4: iRhom2 interactome. Volcano plots quantitatively compare the proteome of co-IPs from control (GFP) with (a) wild type iRhom2 and (b) miR2_W538S based on label-free quantification. Significantly regulated proteins are labelled orange (requirements: p-value < 0.01, difference/ratio: > 4-fold). YWHAB: 14-3-3 beta/alpha; YWHAE: 14-3-3 epsilon; YWHAQ: 14-3-3 zeta. It is worth noting that both wt iRhom2 and miR2_W538S bind FRMD8, a crucial iRhom stability factor, to a similar extent. The volcano plots were generated using Instant Clue [60]. (TIF 1662 KB)

Supplementary file 5: Figure S5: HEK293 cells or iRhom1/iRhom2-deficient MEFs stably expressing the indicated iRhom constructs were utilised. As negative control, cells stably expressing GFP (ctr) were used. (a) ADAM17-mediated shedding activity was assessed by performing a cleavage assay for the AP-tagged substrate IL-1RII in HEK293 cells. Cells were left unstimulated, stimulated with TRAP6 or stimulated with TRAP6 and additionally treated with the ADAM17 inhibitor TAPI1 for 2 h. n = 8. (b), (c) Binding of ADAM17 to the indicated iRhom constructs shown in figure 3a (b) and figure 3b (c) was assessed by densitometric measurements and calculation of the ratio between mADAM17 and the respective iRhom construct. n = 3. (d) ADAM17-mediated shedding activity was assessed in iRhom1/iRhom2-deficient MEFs by performing a cleavage assay for the substrates IL1RII or IL6R tagged with AP. Cells were incubated for 2 h under unstimulated condition, stimulated with the phorbol ester PMA or stimulated with PMA and treated with the inhibitor TAPI1. In case of IL6R, there is a small increase of shedding activity detectable after stimulation with PMA compared to unstimulated conditions in control cells and cells with miR2_W538S. This shedding is not inhibited by TAPI1 and can most likely be attributed to ADAM10 which also sheds IL6R [62]. n = 4. (TIF 1710 KB)

Supplementary file 6: Figure S6: HEK293 cells stably expressing the indicated iRhom constructs or GFP (ctr) were utilised. (a) Cell surface expression of endogenous ADAM10 was measured by flow cytometry. Cells only stained with secondary antibody (ns-ctr) were used as additional control. For quantification, the geometric mean of the fluorescence intensity was determined and normalised to the wt miRhom2 sample. n = 4. (TIF 649 KB)

Supplementary file 7: Figure S7: HEK293 cells stably expressing the indicated iRhom constructs or GFP (ctr) were utilised. (a) – (b) Cell surface expression of iRhom constructs and endogenous ADAM17 was measured by flow cytometry. For quantification, the geometric mean of the fluorescence intensity was determined and normalised to wt hiRhom2 sample, respectively. n = 4. (c) Maturation of ADAM17 depicted in figure 4c was assessed by densitometric measurements and calculation of the ratio between mADAM17 and total ADAM17 derived by the sum of mADAM17 and imADAM17. n = 5. (d) Binding of ADAM17 to the indicated iRhom constructs shown in figure 4c was assessed by densitometric measurements and calculation of the ratio between mADAM17 and the respective iRhom construct. n = 6. (e) Density gradient centrifugation was used to separate Golgi and ER fractions after cell lysis. An anti-GM130 antibody was used to identify cis-Golgi containing fractions. An anti-KDEL antibody was used to label ER-containing fractions. As shown, fraction 2 and 3 only contain cis-Golgi, while fraction 4 to 8 contain both ER and cis-Golgi. While wt miRhom2 is also present in the pure cis-Golgi containing fractions, miR2_W538S is only present in the ER/Golgi mixed fractions. (TIF 2224 KB)

Supplementary file 8: Figure S8: (a) The folding process of proteins or their ER-luminal domains is closely monitored by the ER quality control system, which assists the folding process and checks whether domains fold correctly. One of the first steps of this ER quality control for N-glycosylated proteins is binding to either calnexin or calreticulin, both of which are lectin chaperones. Correctly folded proteins can rapidly exit the calnexin-mediated quality control system and enter the ER exit sites (ERES) [37]. In contrast, misfolded or unfolded proteins undergo multiple calnexin-mediated attempts to achieve correct folding. Strong calnexin binding therefore indicates a folding problem within the protein. In contrast, weak binding indicates rapid passage through the calnexin-dependent ER quality control and indicates a correctly folded protein. If multiple folding attempts do not result in correct folding, the misfolded or unfolded target protein is subjected to a rapid ERAD. Another hallmark of a folding problem within an ER-luminal part of a protein is therefore reduced stability and a drastically shortened half-life. (b) HEK293 cells stably expressing the indicated HA-tagged iRhom deletions were additionally transfected with myc-tagged calnexin. Co-IP experiments with the different iRhom2 constructs as bait were performed and analysed by western blotting using a α-myc antibody to probe for calnexin. Immunoblot signals were quantified and calnexin binding was determined by calculation of the signal ratio between calnexin and the respective iRhom2 construct. n = 3. The deletion of the whole IRHD left a protein (miR2_ΔIRHD) with no luminal domain. Hence, no calnexin binding would be expected for this deletion. (TIF 1964 KB)

Supplementary file 9: Figure S9: (a) – (b) MEFs, deficient for iRhom1 and iRhom2 and instead stably expressing the indicated iRhom constructs were used for the described experiments. As negative control, cells stably expressing GFP (ctr) studied in parallel. To analyse the stability of the iCERES mutant miR2_3 compared to wt miRhom2 a cycloheximide-based pulse chase experiment was performed. n = 3. (a) Samples were collected at the indicated time points after starting treatment with cycloheximide (CHX) and were then subjected co-immunoprecipitations and ConA precipitations with subsequent immunoblotting. Immunoblot were analysed for iRhom2 constructs and ADAM17. (b) Immunoblot signals (a) were quantified and normalised to samples without CHX at time point 0 h (Ø). At time point 0 the protein amount was set to 1. (c) - (d) HEK293 cells stably expressing the indicated iRhom constructs or GFP (ctr) were utilised. (c) To inhibit lysosomal degradation, cells were treated as indicated either with or without 500 nM Bafilomycin A1 (Baf) for 4 h or 8 h. iRhom constructs were immunoprecipitated (IP) and immunoblotted. (d) Immunoblot signals representing ER-glycosylated (green arrow) or Golgi-glycosylated forms (red arrow) of the indicated iRhom2 constructs were assessed by densitometric measurements and calculation of the ratio between ER-glycosylated and Golgi-glycosylated forms. n = 3. (e) - (f) MEFs, deficient for iRhom1 and iRhom2, stably expressing the indicated iRhom constructs were used for the described experiments. As negative control, cells stably expressing GFP (vector ctr) were used. To inhibit lysosomal degradation, cells were treated as indicated either with or without 500 nM Bafilomycin A1 (Baf) for 8 h. (e) To analyse ADAM17 maturation, glycosylated proteins were enriched by concanavalin A beads (ConA). (f) Maturation of ADAM17 depicted in (e) was assessed by densitometric measurements and calculation of the ratio between mADAM17 and total ADAM17 derived by the sum of mADAM17 and imADAM17. n = 3. (TIF 2204 KB)

Supplementary file 10: Figure S10: (a) Expression level of ADAM17, iRhom1 and iRhom2 mRNA in liver, lung and spleen from iRhom2 knock out mice (Del) was measured by qPCR. Since the used primers for the qPCR bind a region distant from the mutated site, also mRNA of the Del mice could be detected. n = 3. (b) Protein expression of endogenous iRhom2 and ADAM17 in indicated BMDMs was detected by precipitating glycosylated proteins with concanavalin A beads and immunoblotting. Transferrin receptor (TfR) served as input control. BMDMs were derived either from mice homozygous for iRhom knock out (Del), heterozygous or wild type littermates (wt), respectively. n = 3. (c) – (e) Binding of indicated iRhom2 construct to the indicated interactor depicted in figure 8e (c), figure 8f (d) and figure 8g (e) was assessed by densitometric measurements and calculation of the ratio between indicated interactor and the respective iRhom construct. n = 3. (TIF 1441 KB)

Supplementary file11 (XLSX 5241 KB)

Supplementary file12 (XLSX 92 KB)

Supplementary file13 (PDF 3016 KB)

## Data Availability

The mass spectrometry proteomics data have been deposited to the ProteomeXchange Consortium (http://proteomecentral.proteomexchange.org) via the PRIDE [1] partner repository with the dataset identifier PXD016948. The login and the password for reviewers are reviewer92598@ebi.ac.uk and DXc4QSOP. (http://www.ebi.ac.uk/pride/archive/projects/PXD016948).

## Abbreviations

ADAM: A disintegrin and metalloproteinases
AREG: Amphiregulin
Co-IP: Co-Immunoprecipitation
EGFR: Epidermal growth factor receptor
ERES: ER exit sites
ERGIC: ER-Golgi intermediate compartment
iCERES: iRhom Conserved ER to Golgi Export Sequence
HBEGF: Heparin-binding EGF-like growth factor
IL1R_II_: Interleukin 1 receptor type II
IL6: Interleukin 6
SNARE: Soluble NSF attachment proteins receptors
IRHD: iRhom homology domain
STX: Syntaxin
TGFα: Transforming growth factor alpha
TNFα: Tumour necrosis factor alpha
