# Supplementary material for: The iRhom homology domain is indispensable for ADAM17-mediated TNFα and EGF receptor ligand release
Source: Cell Mol Life Sci. 2021 May 5;78(11):5015–40. doi: 10.1007/s00018-021-03845-3 (PMC8233286; doi:10.1007/s00018-021-03845-3)

**List of used mice:**

| ID | Sex | Strain | Grade | Birth | Parents (ID) |
|---|---|---|---|---|---|
| 64S-G3625 | f | W538S | hom | 27.01.2019 | 64U-81128 (m) / 64U-81129 (f) |
| 64S-G3626 | f | W538S | wt | 27.01.2019 | 64U-81128 (m) / 64U-81129 (f) |
| 64S-G3627 | f | W538S | hom | 27.01.2019 | 64U-81128 (m) / 64U-81129 (f) |
| 64S-G3628 | f | W538S | wt | 27.01.2019 | 64U-81128 (m) / 64U-81129 (f) |
| 64S-G4606 | m | W538S | wt | 06.02.2019 | 64U-72654 (m) / 64U-72657 (f) / 64U-72658 (f) |
| 64S-G4607 | m | W538S | hom | 06.02.2019 | 64U-72654 (m) / 64U-72657 (f) / 64U-72658 (f) |
| 64U-80966 | m | W538S | hom | 09.09.2018 | 64S-E4974 (m) / 64U-76100 (f) |
| 64U-80967 | m | W538S | hom | 09.09.2018 | 64S-E4974 (m) / 64U-76100 (f) |
| 64U-80973 | f | W538S | wt | 09.09.2018 | 64S-E4974 (m) / 64U-76100 (f) |
| 64U-81116 | m | W538S | wt | 12.09.2018 | 64S-E4973 (m) / 64U-76096 (f) |
| 64U-81122 | f | W538S | wt | 12.09.2018 | 64S-E4973 (m) / 64U-76096 (f) |
| 64U-81123 | f | W538S | hom | 12.09.2018 | 64S-E4973 (m) / 64U-76096 (f) |
| 64S-G3873 | m | iR2KO/Del | hom | 29.01.2019 | 64U-75714 (m) / 64U-75716 (f) / 64U-75717 (f) |
| 64S-G3877 | m | iR2KO/Del | hom | 29.01.2019 | 64U-75714 (m) / 64U-75716 (f) / 64U-75717 (f) |
| 64S-G3879 | f | iR2KO/Del | wt | 29.01.2019 | 64U-75714 (m) / 64U-75716 (f) / 64U-75717 (f) |
| 64S-G3880 | f | iR2KO/Del | wt | 29.01.2019 | 64U-75714 (m) / 64U-75716 (f) / 64U-75717 (f) |
| 64S-G8777 | m | iR2KO/Del | hom | 22.02.2019 | 64U-75714 (m) / 64U-75716 (f) / 64U-75717 (f) |
| 64S-G8781 | f | iR2KO/Del | wt | 22.02.2019 | 64U-75714 (m) / 64U-75716 (f) / 64U-75717 (f) |

C57Bl/6J background; iRhom2 KO (Del13) and iRhom2_W538S mutation and respective wild type and heterozygous littermates; male and females were used; 10 - 18 weeks old

Figure 1C

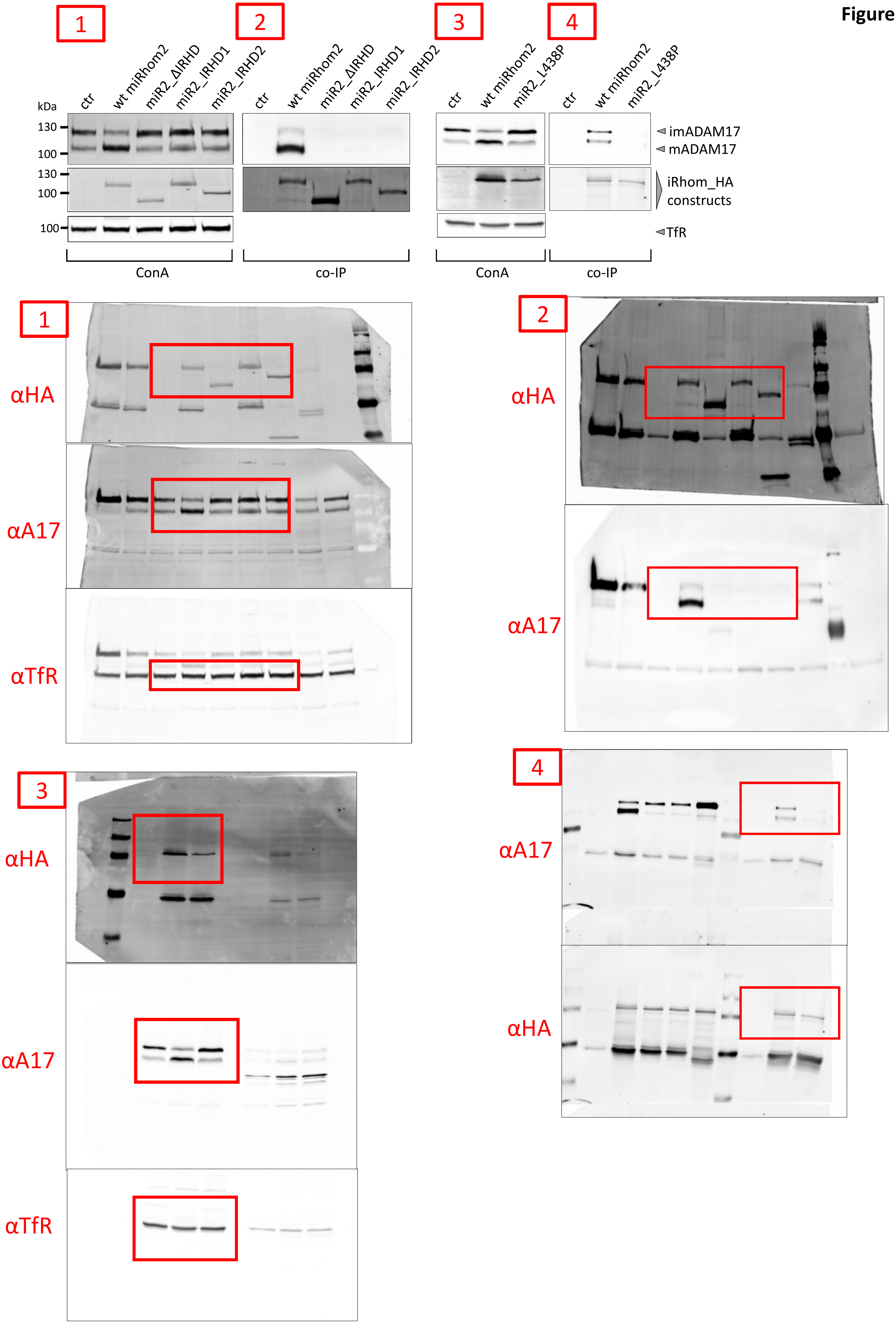
1
2
3
4
kDa
ctr
wt miRhom2
miR2_ΔIRHD
miR2_IRHD1
miR2_IRHD2
ctr
wt miRhom2
miR2_ΔIRHD
miR2_IRHD1
miR2_IRHD2
ctr
wt miRhom2
miR2_L438P
ctr
wt miRhom2
miR2_L438P
130
100
130
100
100
imADAM17
mADAM17
iRhom_HA constructs
TfR
ConA
co-IP
ConA
co-IP
1
αHA
αA17
αTfR
2
αHA
αA17
3
αHA
αA17
αTfR
4
αA17
αHA

**Figure 2B**

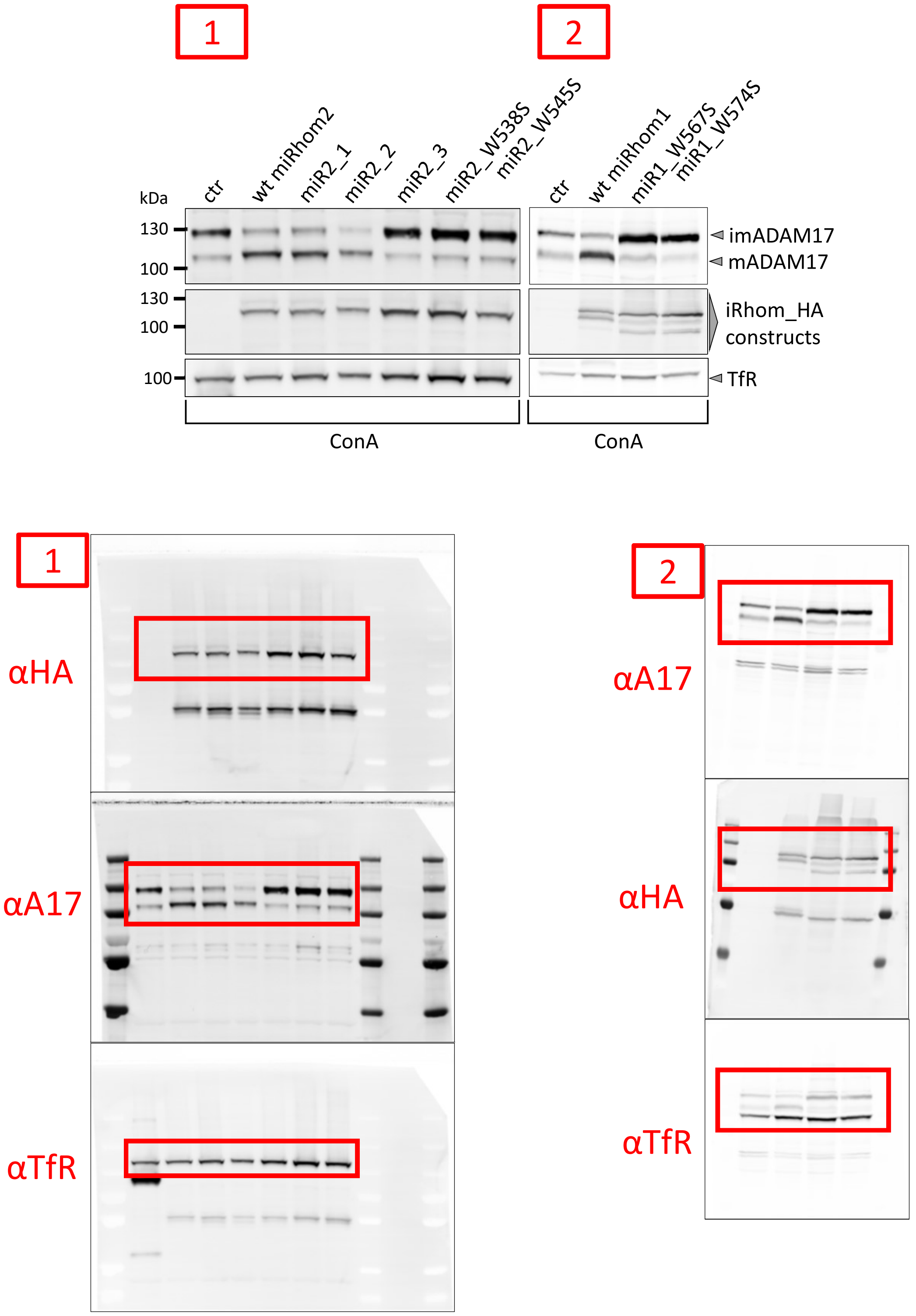
1
2
ctr
wt miRhom2
miR2_1
miR2_2
miR2_3
miR2_W538S
miR2_W545S
ctr
wt miRhom1
miR1_W567S
miR1_W574S
kDa
130
100
130
100
100
imADAM17
mADAM17
iRhom_HA
constructs
TfR
ConA
ConA
1
αHA
αA17
αTfR
2
αA17
αHA
αTfR

**Figure 2C**

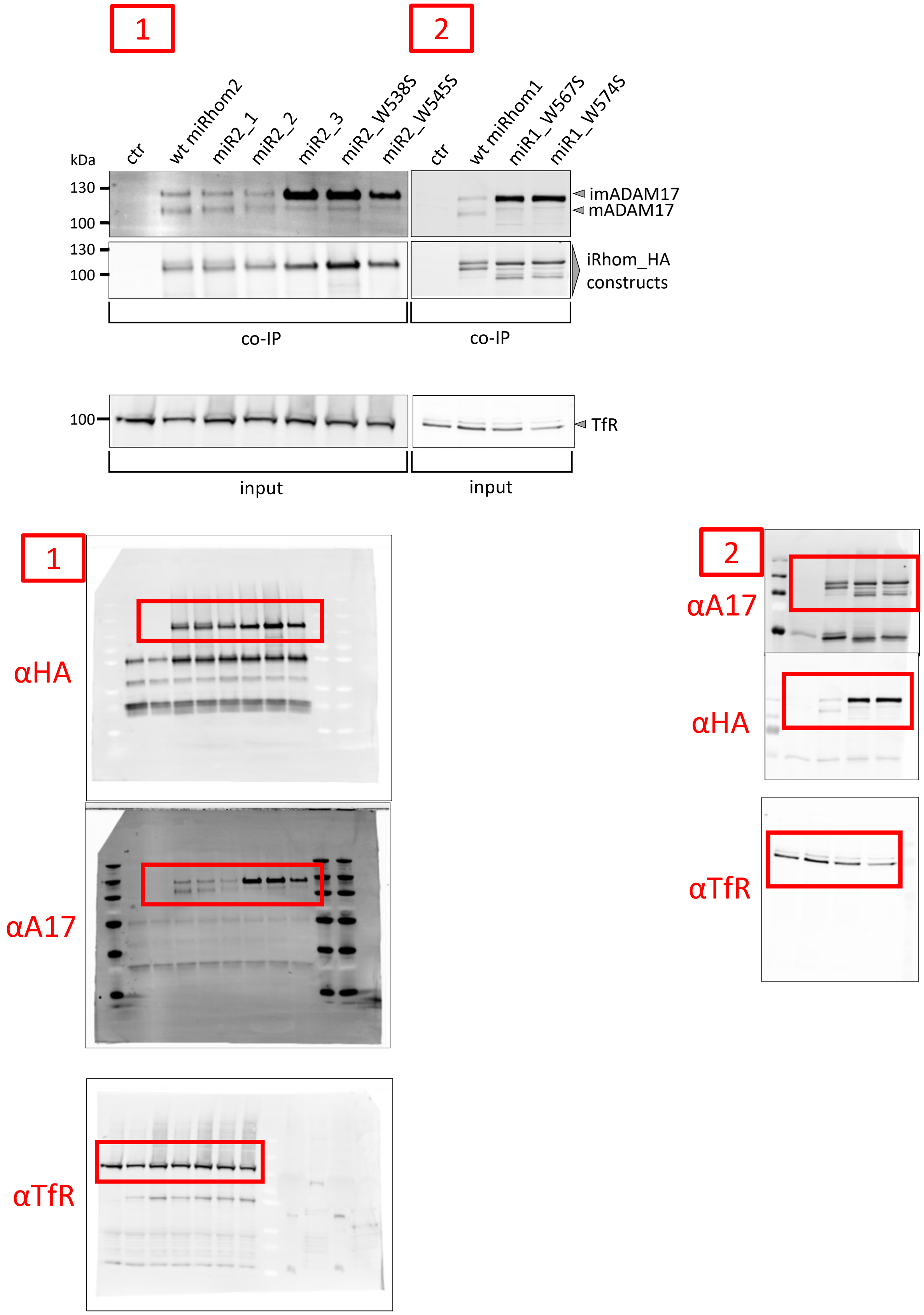
1
2
kDa
ctr
wt miRhom2
miR2_1
miR2_2
miR2_3
miR2_W538S
miR2_W545S
ctr
wt miRhom1
miR1_W567S
miR1_W574S
130
100
130
100
imADAM17
mADAM17
iRhom_HA constructs
co-IP
co-IP
100
TfR
input
input
1
αHA
αA17
αTfR
2
αA17
αHA
αTfR

**Figure 3A**

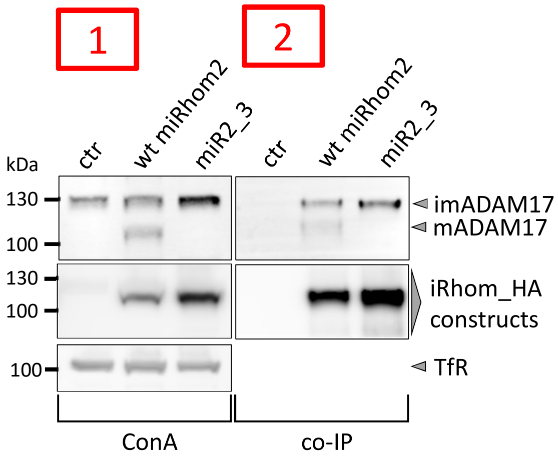
1
2
kDa
ctr
wt miRhom2
miR2_3
ctr
wt miRhom2
miR2_3
130
100
130
100
100
imADAM17
mADAM17
iRhom_HA
constructs
TfR
ConA
co-IP

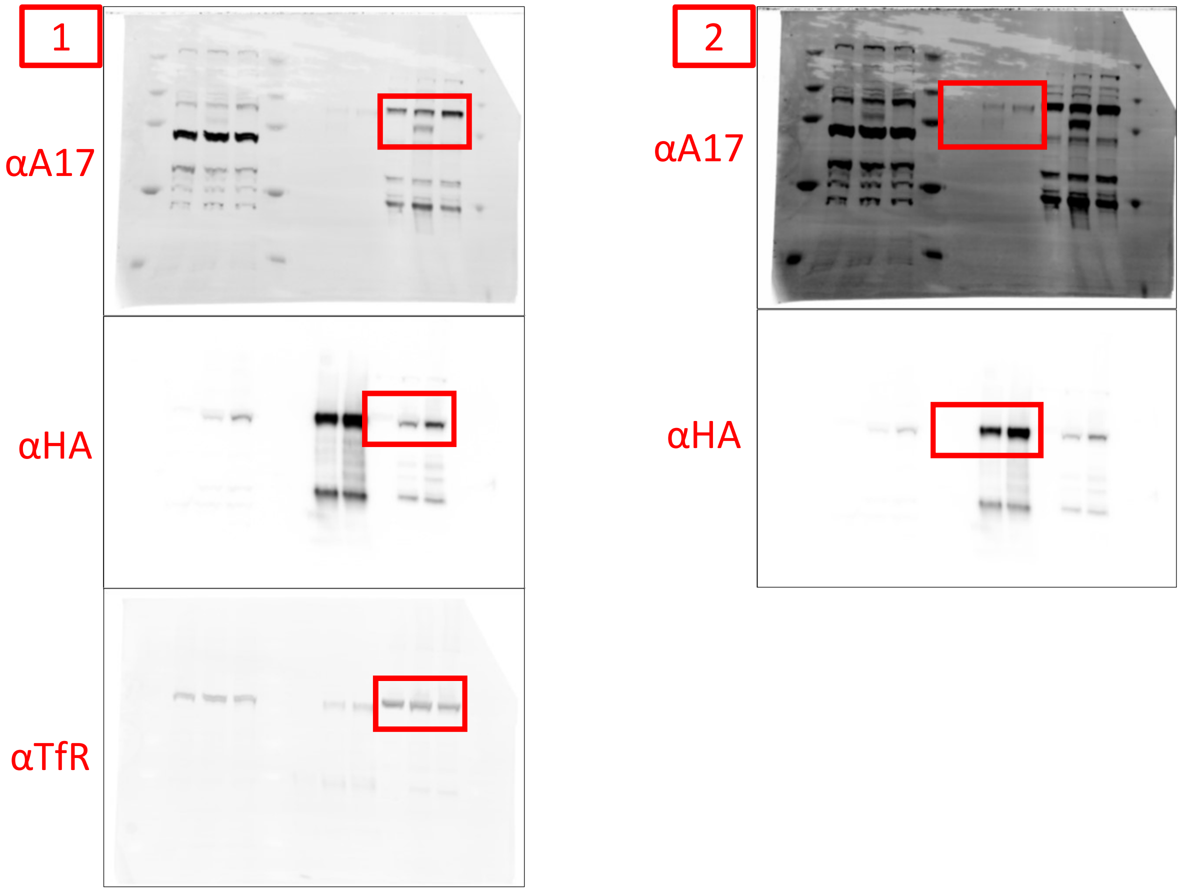
1
αA17
αHA
αTfR
2
αA17
αHA

# Figure 3B

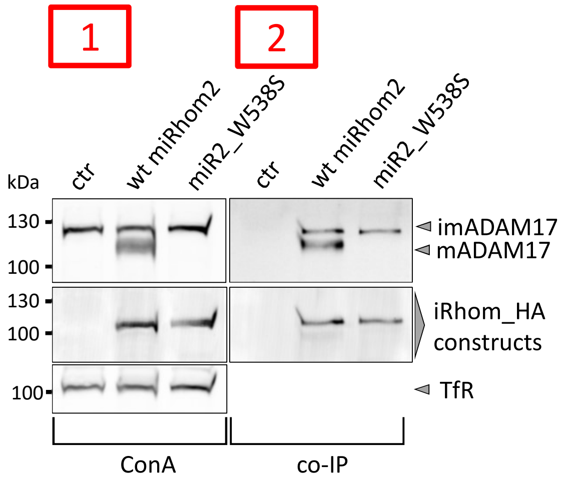
1
2
kDa
ctr
wt miRhom2
miR2_W538S
ctr
wt miRhom2
miR2_W538S
130
100
130
100
100
imADAM17
mADAM17
iRhom_HA
constructs
TfR
ConA
co-IP

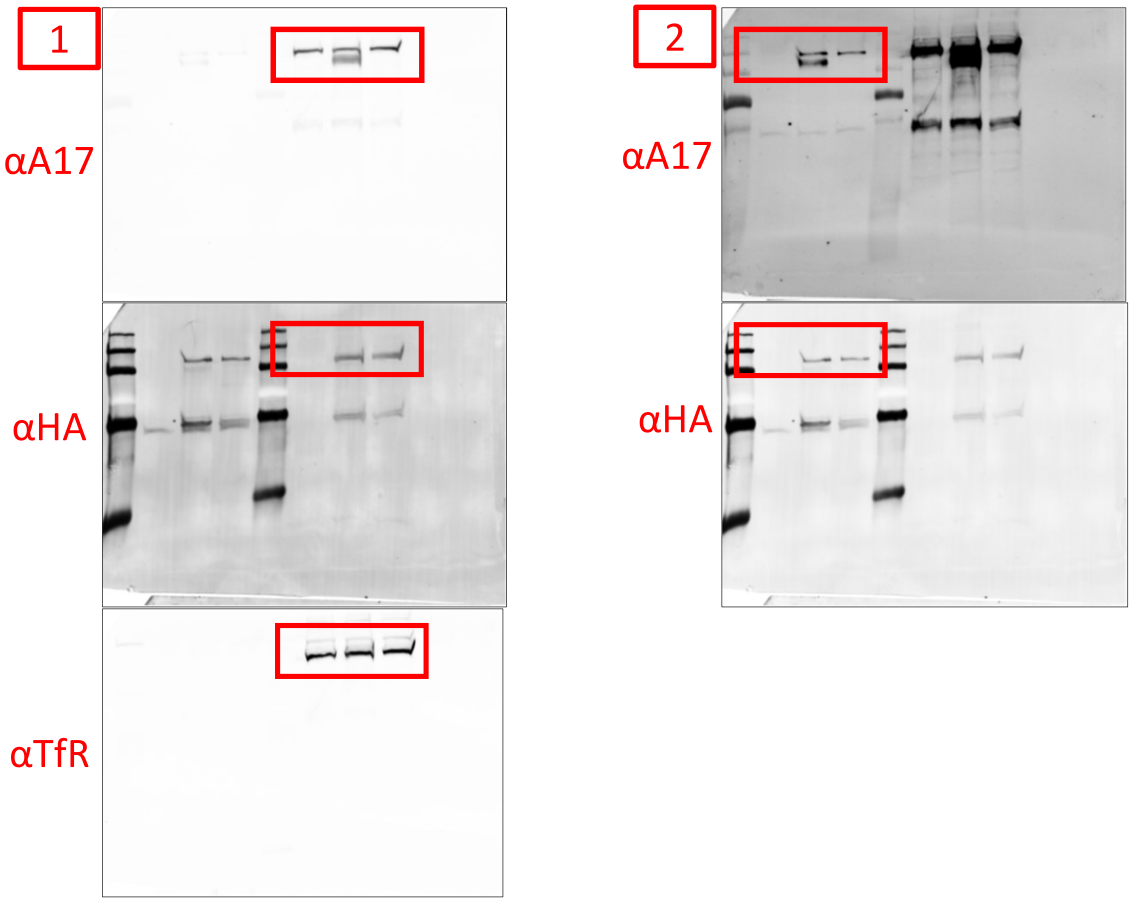
1
αA17
αHA
αTfR
2
αA17
αHA

**Figure 4B**

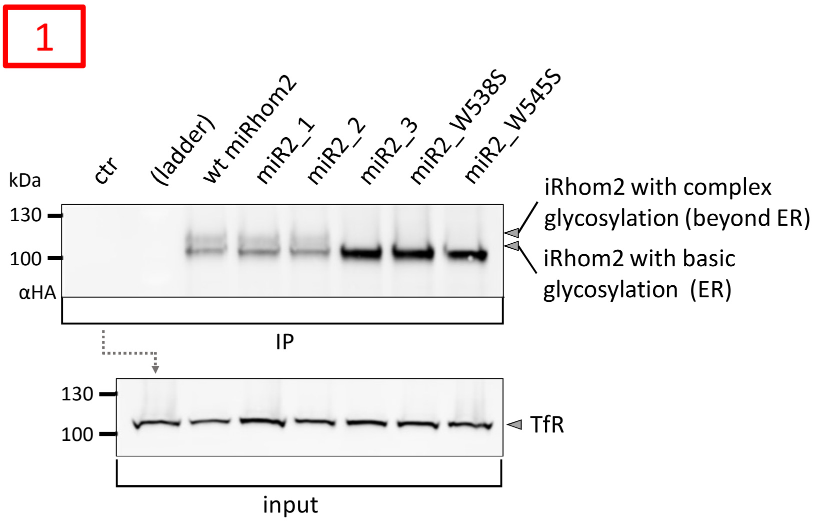


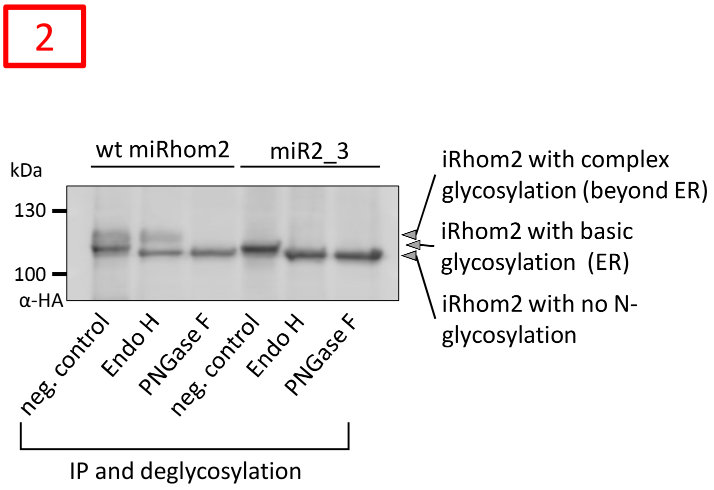


## N = 1

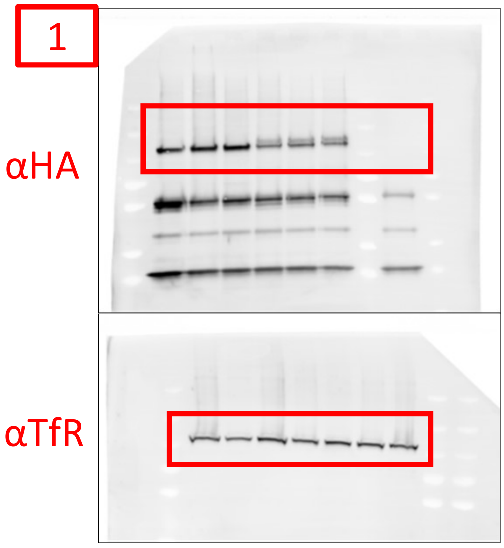


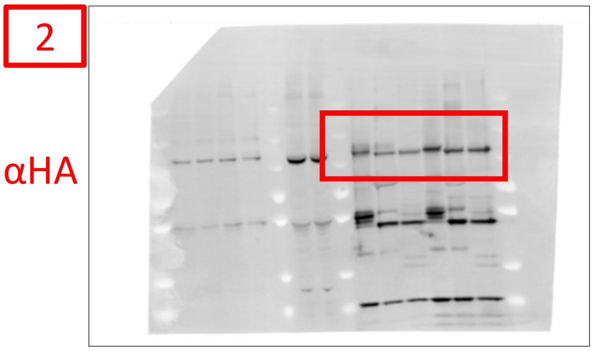


## N = 2

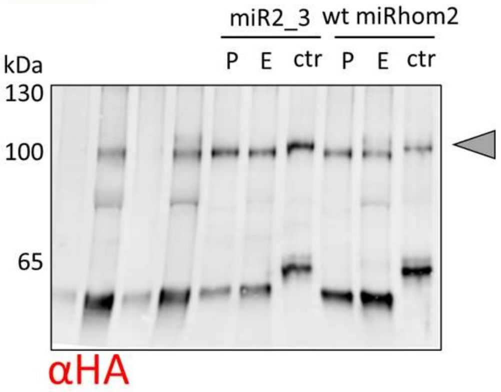


## N = 3

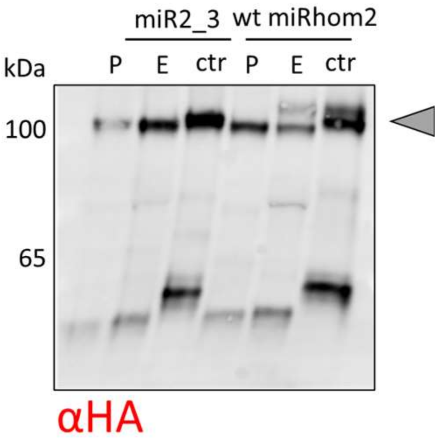

**Figure 4C**

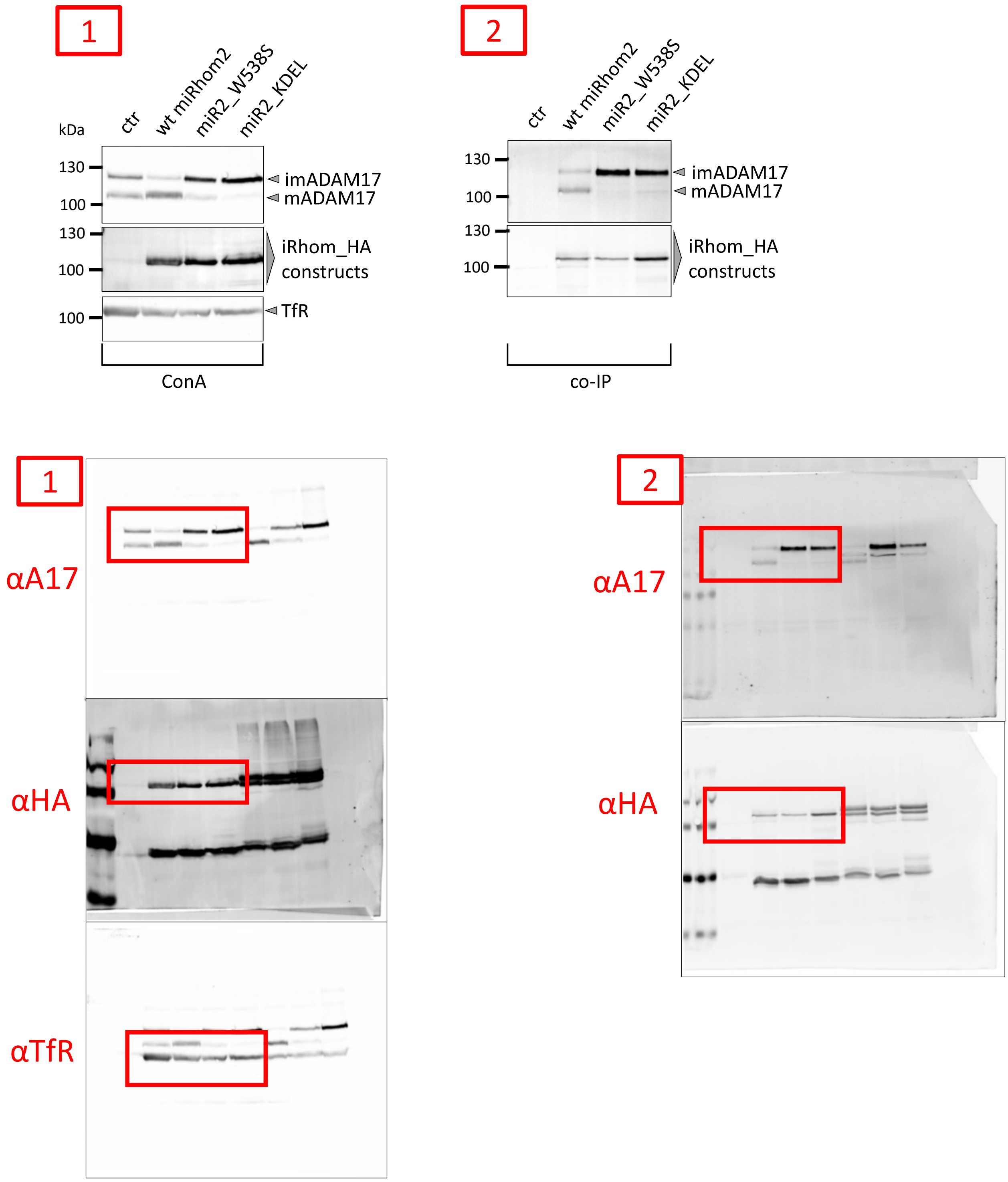
1
ctr
wt miRhom2
miR2_W538S
miR2_KDEL
kDa
130
100
imADAM17
mADAM17
iRhom_HA constructs
TfR
ConA
2
co-IP
αA17
αHA
αTfR

**Figure 5A**

1

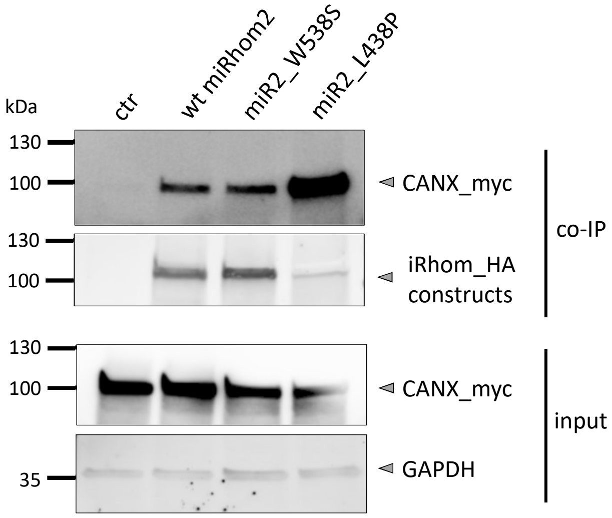


1

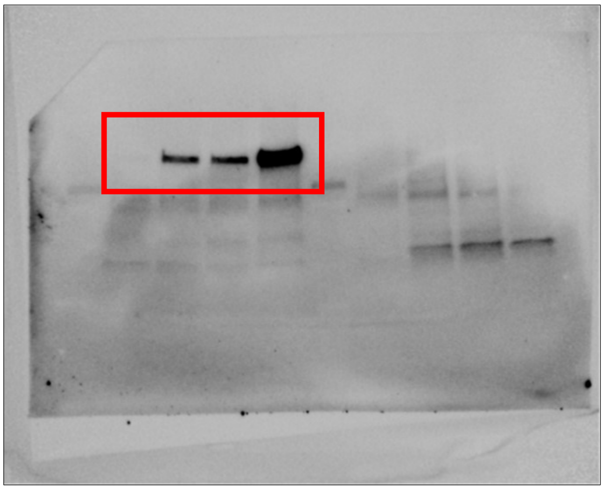

coIP αMyc_CANX

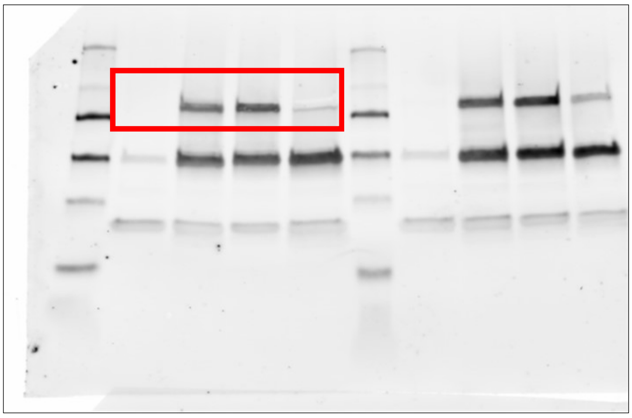

coIP αHA

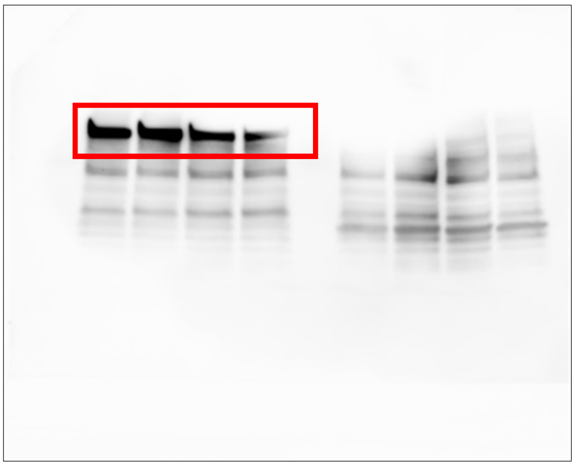

input αMyc_CANX

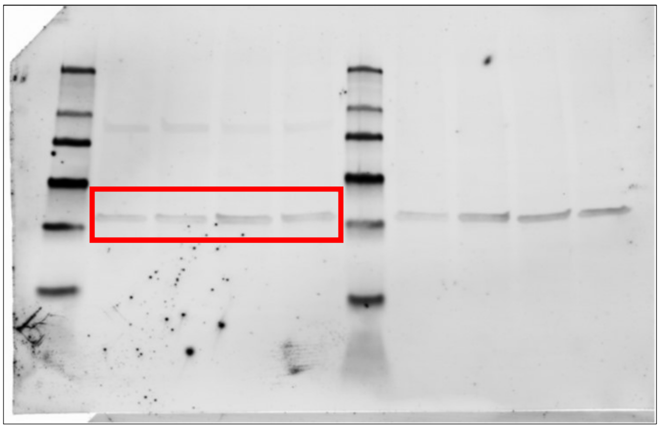

input αGAPDH

**Figure 5C**

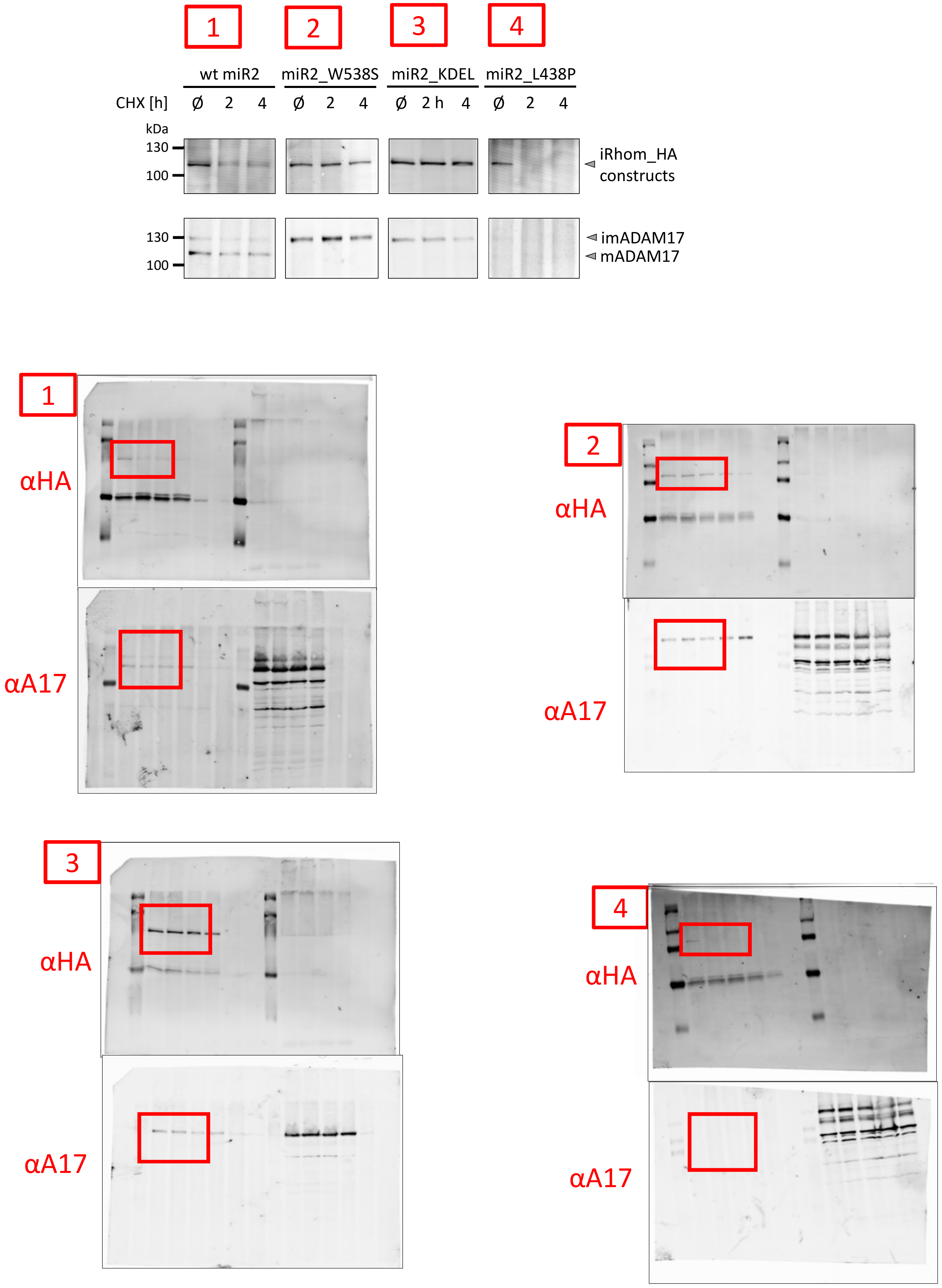
1
2
3
4
wt miR2
miR2_W538S
miR2_KDEL
miR2_L438P
CHX [h]
Ø 2 4 Ø 2 4 Ø 2 h 4 Ø 2 4
kDa
130
100
iRhom_HA constructs
130
100
imADAM17
mADAM17
1
αHA
αA17
2
αHA
αA17
3
αHA
αA17
4
αHA
αA17

**Figure 7B**

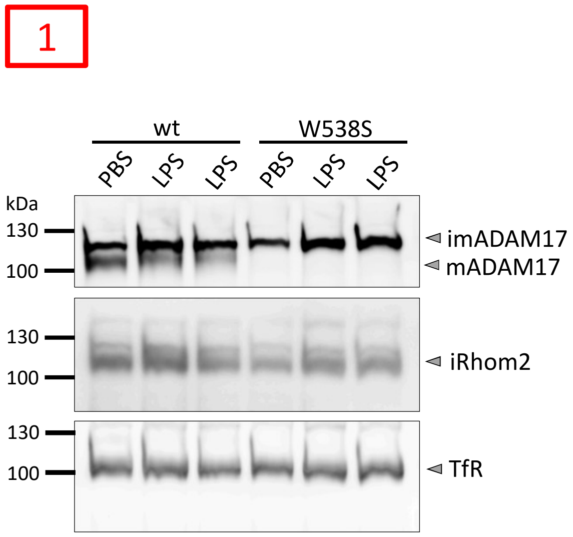

1
wt
W538S
PBS
LPS
LPS
PBS
LPS
LPS
kDa
130
100
imADAM17
mADAM17
130
100
iRhom2
130
100
TfR


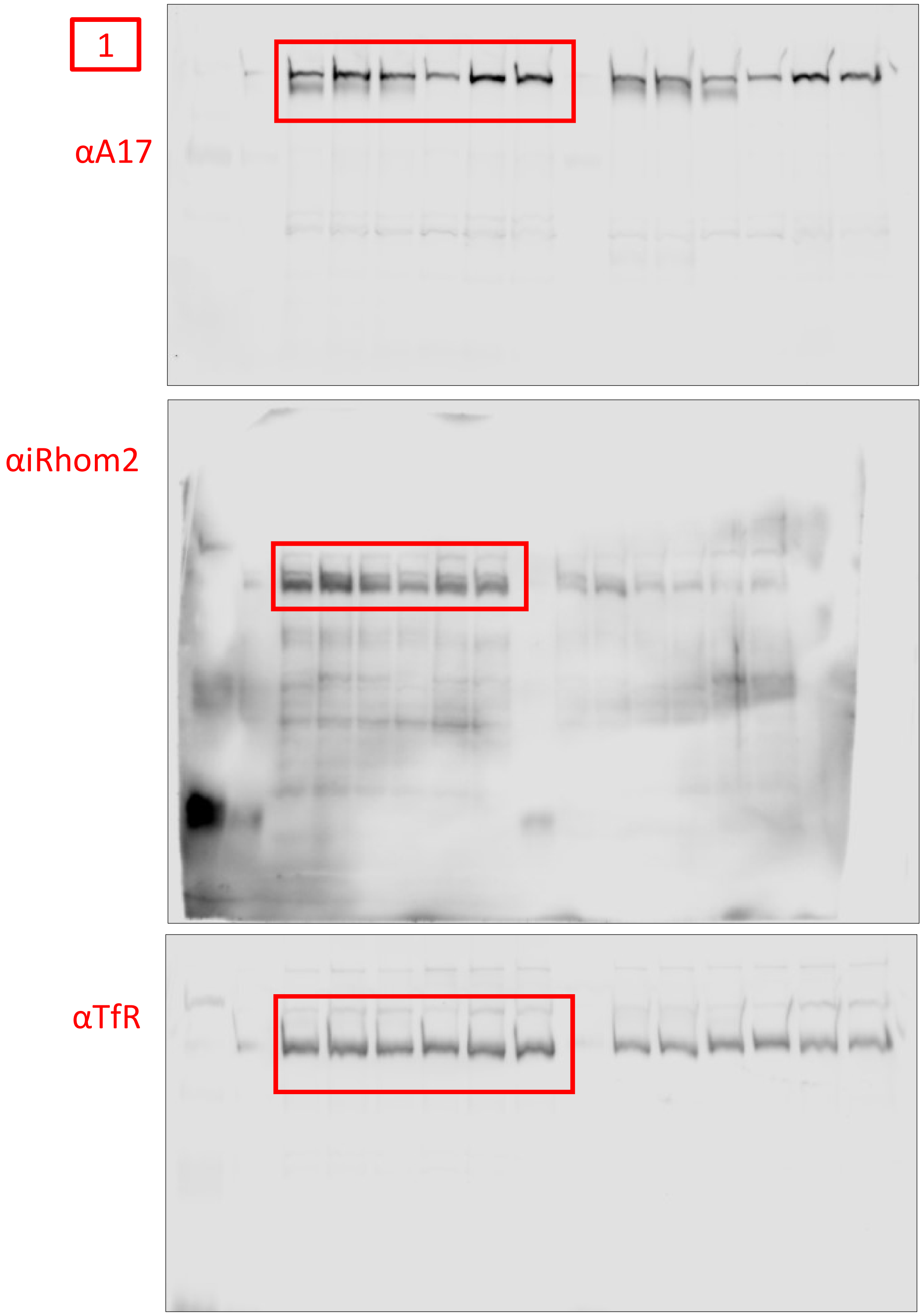

1
αA17
αiRhom2
αTfR

**Figure 8E**

1

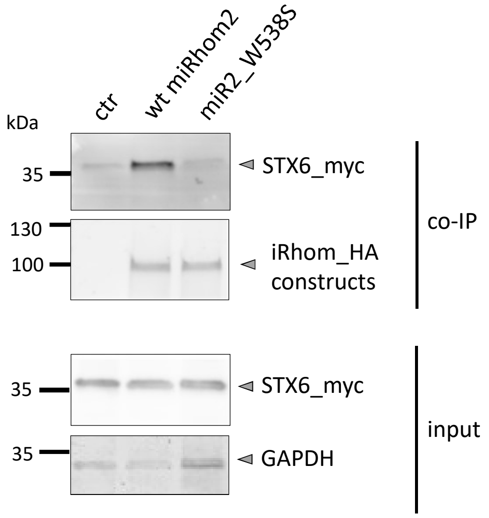


1

coIP αMyc_ STX6

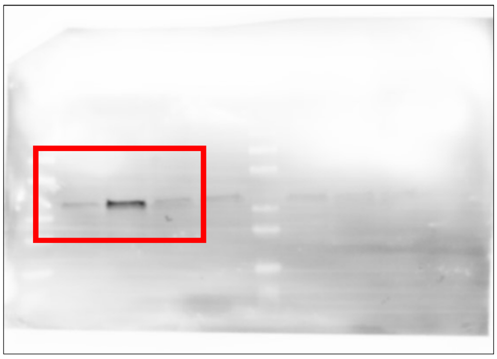

coIP αHA

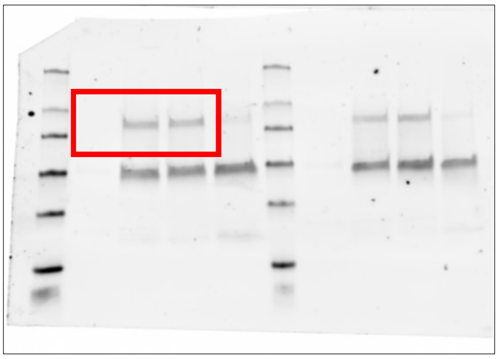

input αMyc_ STX6

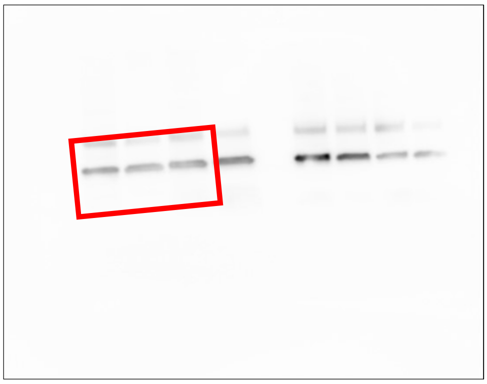

Input GAPDH

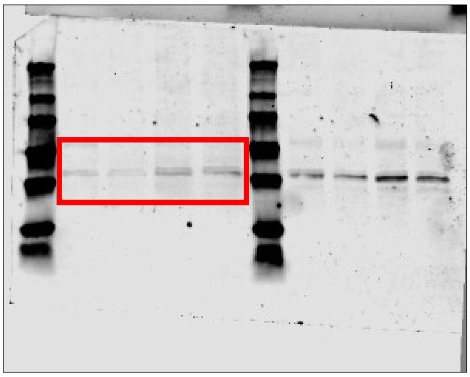

**Figure 8D**

1

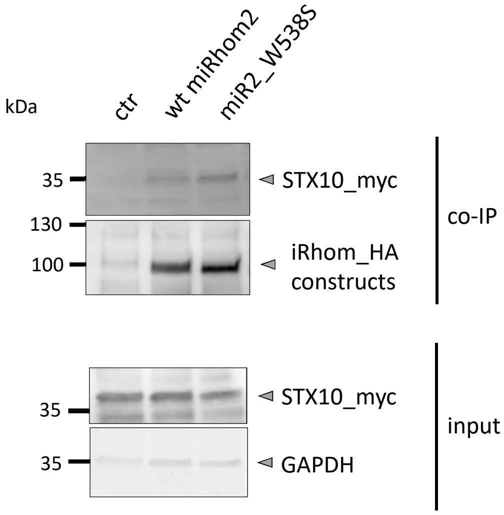


1

coIP αMyc_ STX10

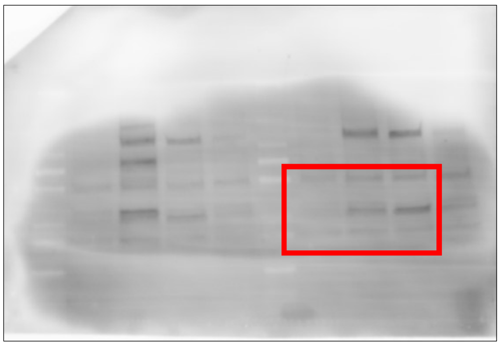

coIP αHA

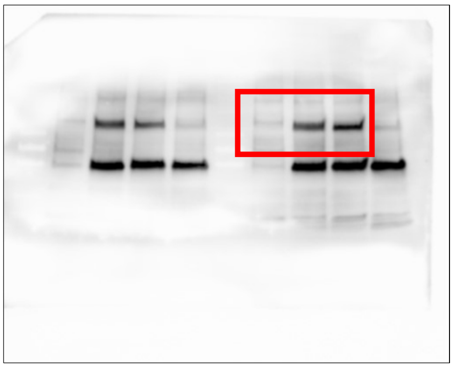

input αMyc_ STX10

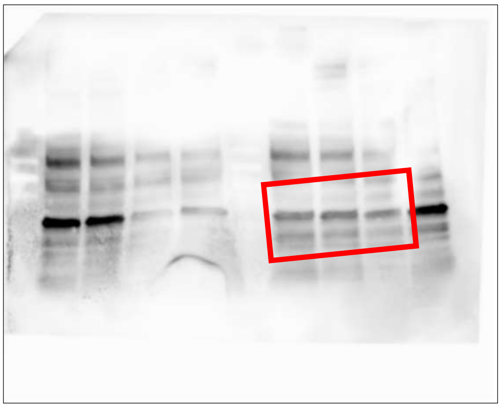

Input GAPDH

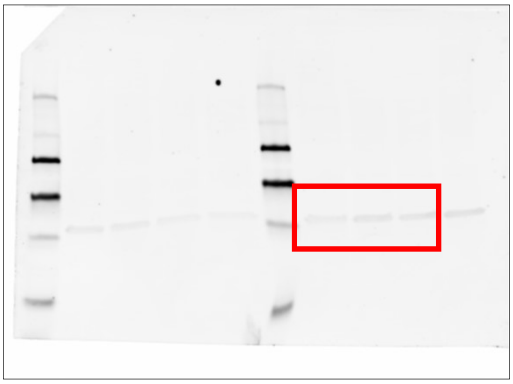

**Figure 8F**

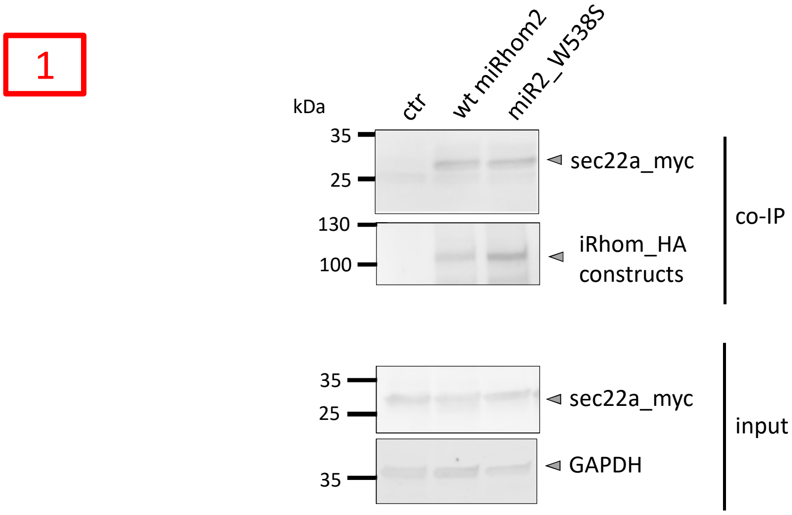
1
kDa
ctr
wt miRhom2
miR2_W538S
35
25
130
100
sec22a_myc
iRhom_HA
constructs
co-IP
35
25
sec22a_myc
35
GAPDH
input

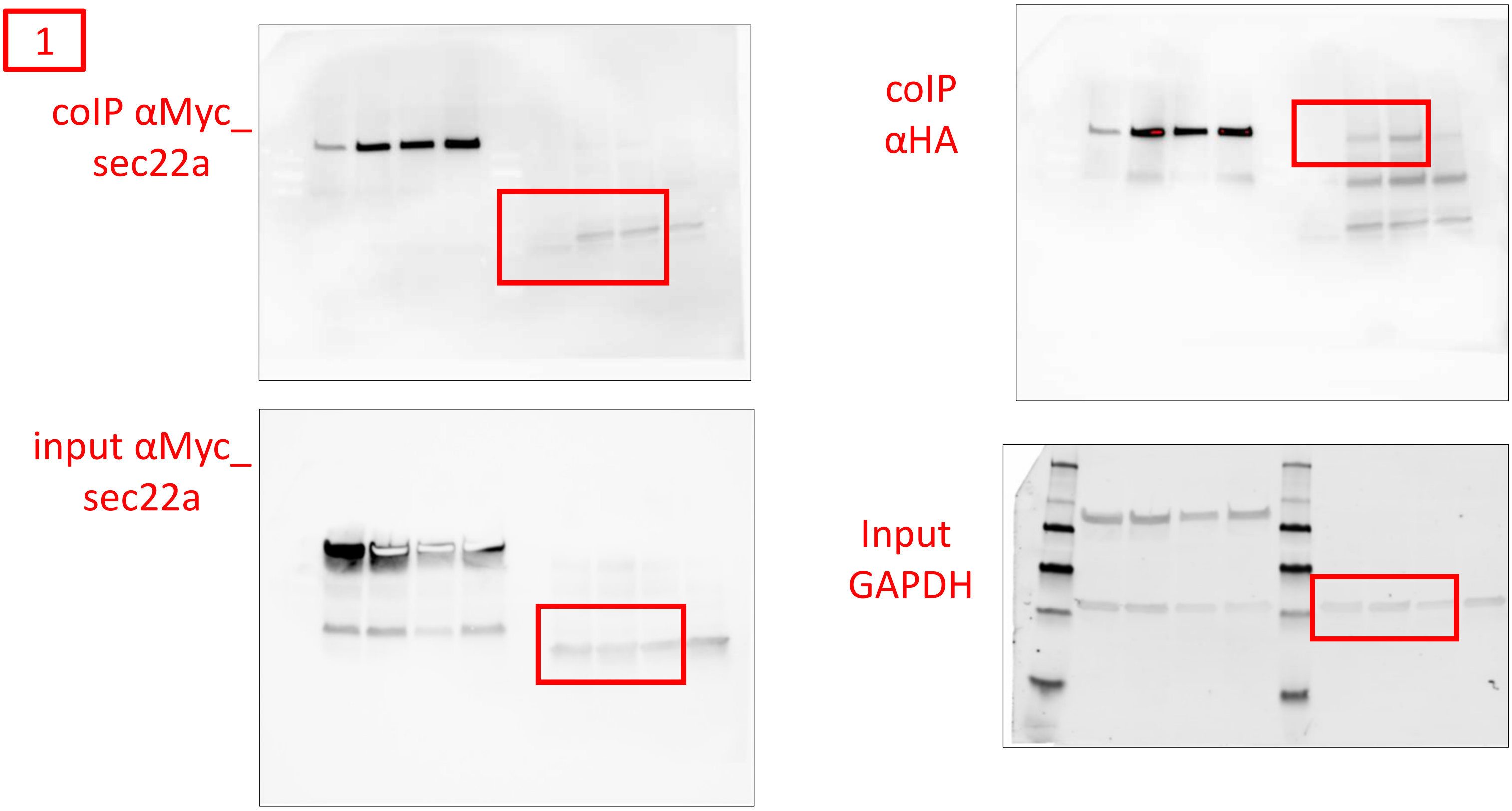
1
coIP αMyc_
sec22a
coIP
αHA
input αMyc_
sec22a
Input
GAPDH

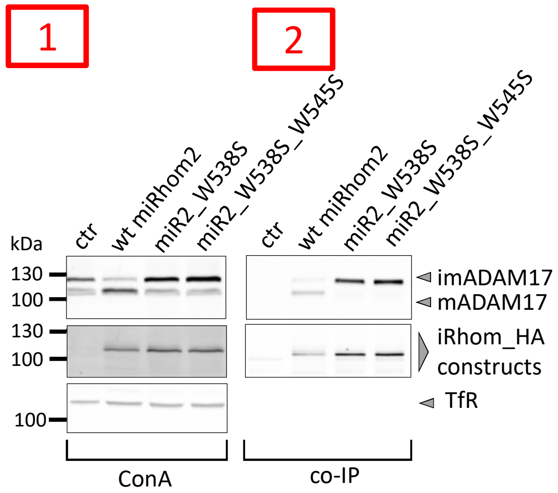
1
2
kDa
ctr
wt miRhom2
miR2_W538S
miR2_W538S_W545S
ctr
wt miRhom2
miR2_W538S
miR2_W538S_W545S
130
100
130
100
100
imADAM17
mADAM17
iRhom_HA
constructs
TfR
ConA
co-IP

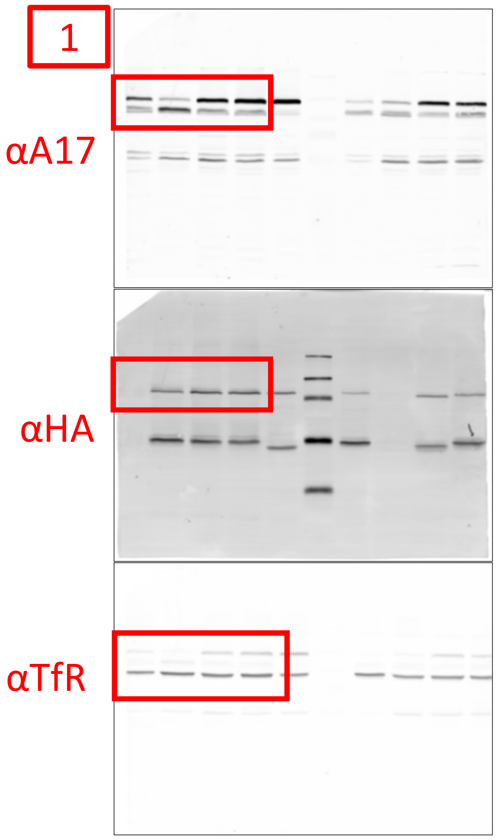
1
αA17
αHA
αTfR

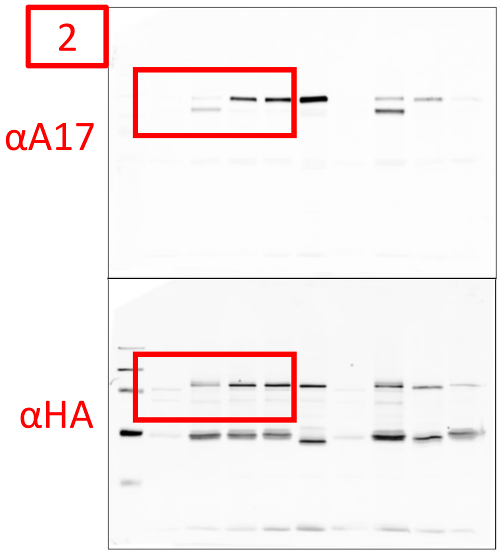
2
αA17
αHA

**Supplementary Figure S3D**

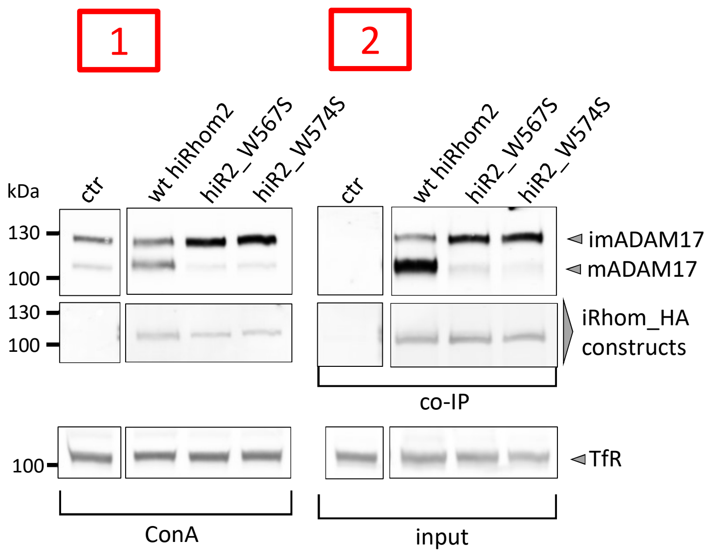


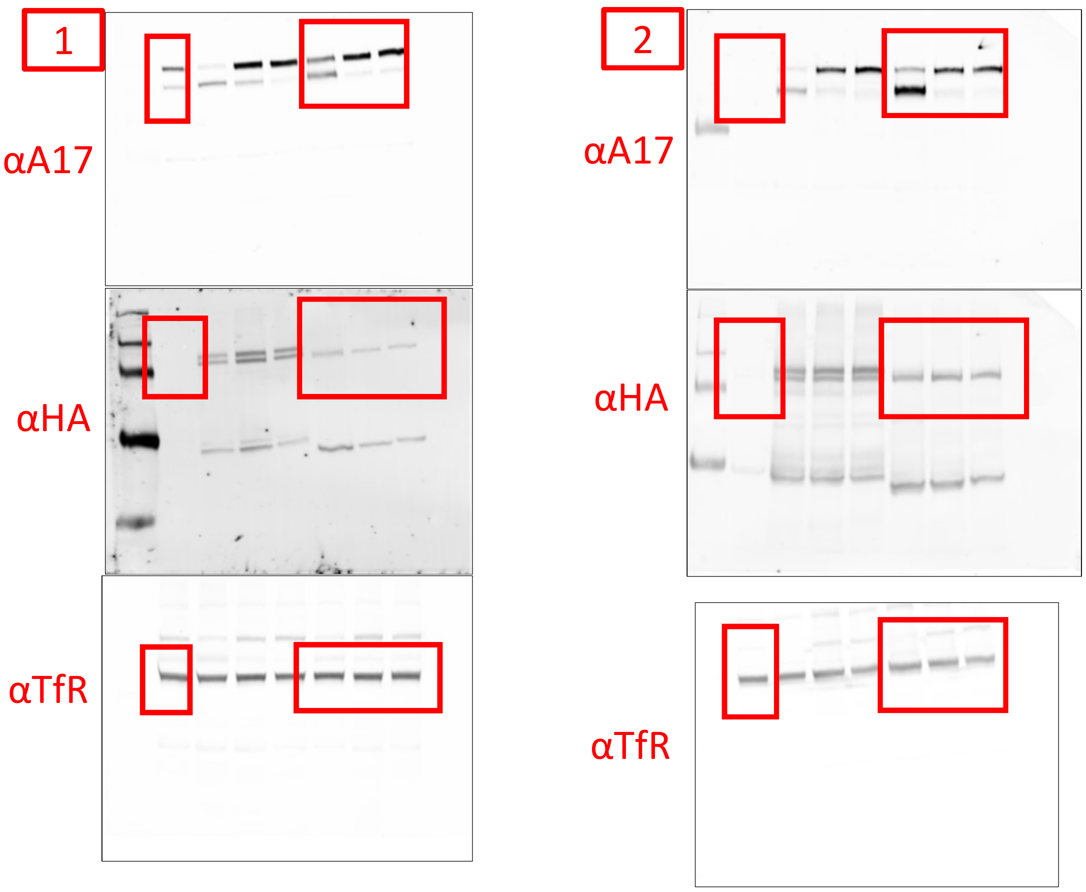

## Supplementary Figure S7E

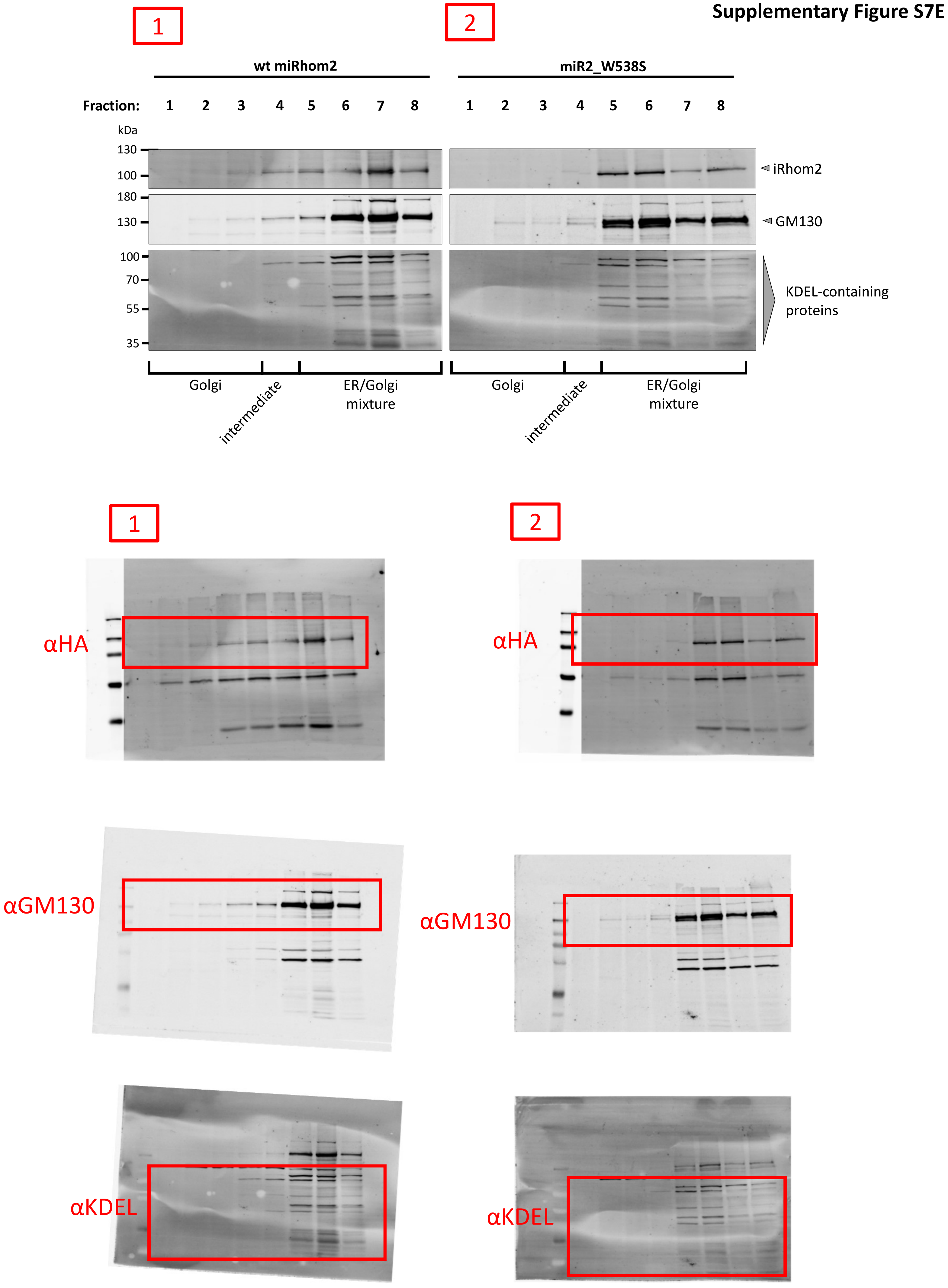

**Supplementary Figure S8B**

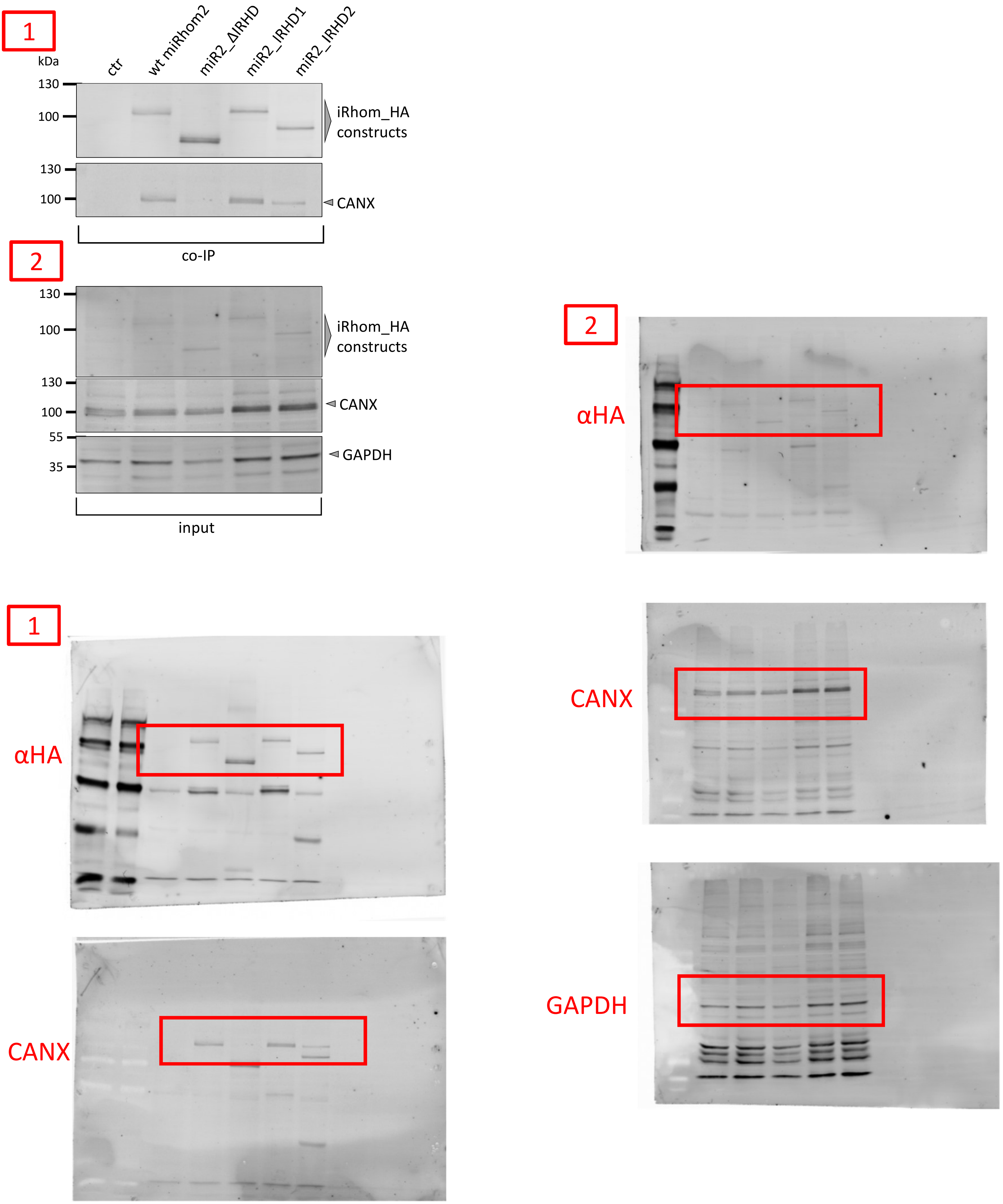

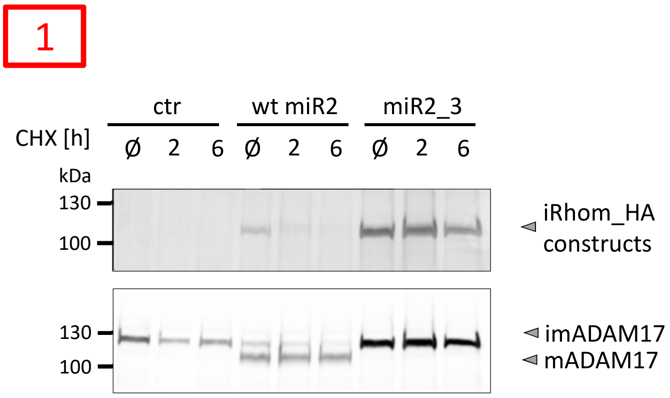
1
ctr
wt miR2
miR2_3
CHX [h]
Ø 2 6 Ø 2 6 Ø 2 6
kDa
130
100
iRhom_HA constructs
130
100
imADAM17
mADAM17

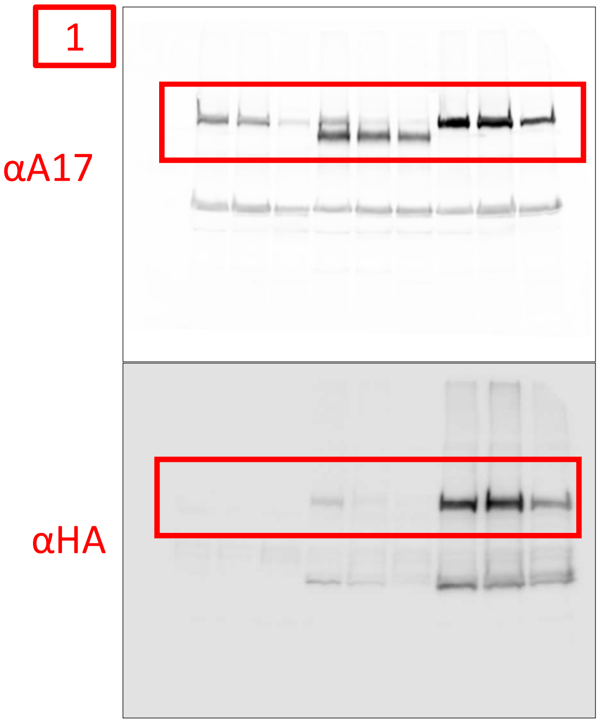
1
αA17
αHA

**Supplementary Figure S9C**

1

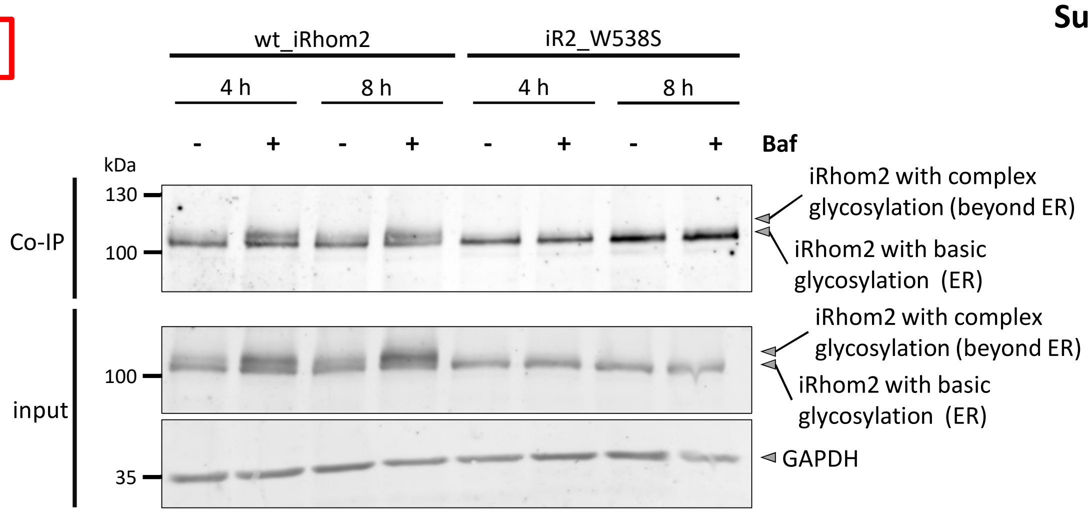


1

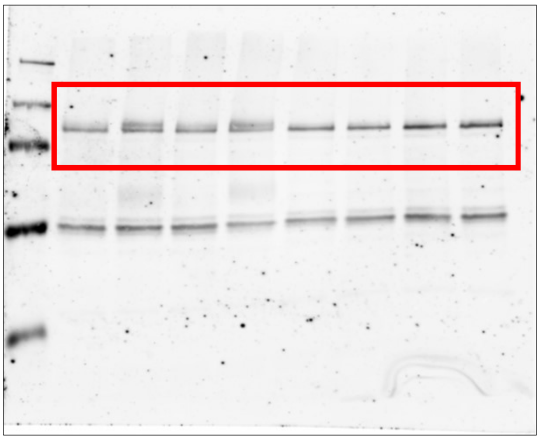

αHA

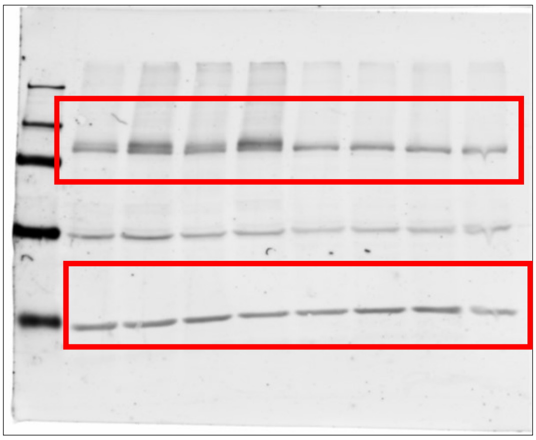

αHA

αGAPDH

Supplementary Figure S9e

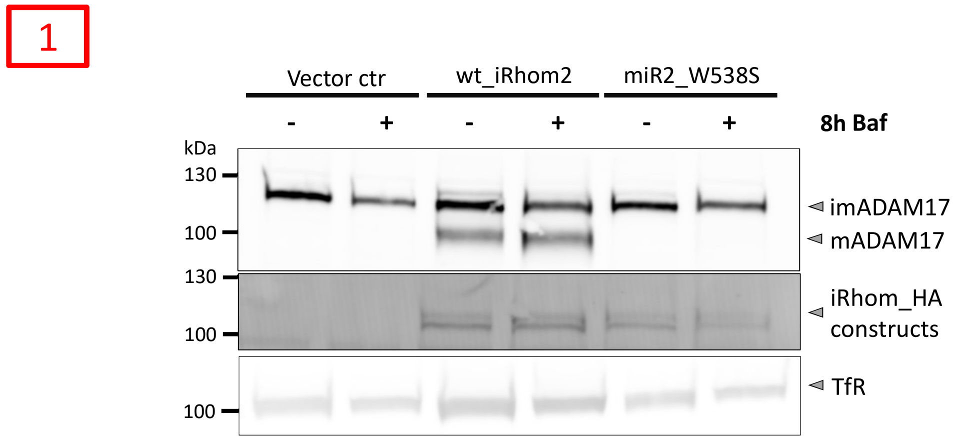


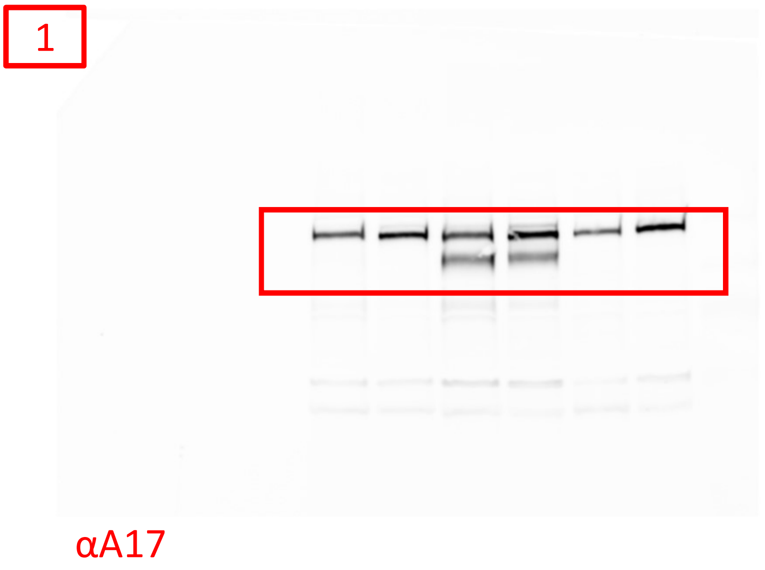


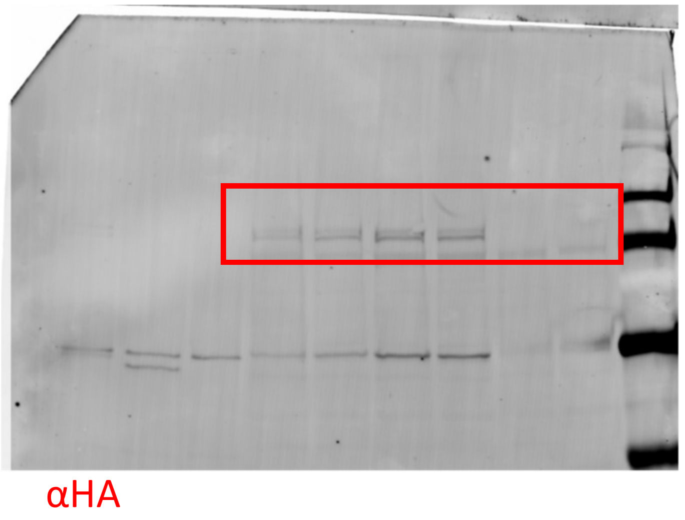


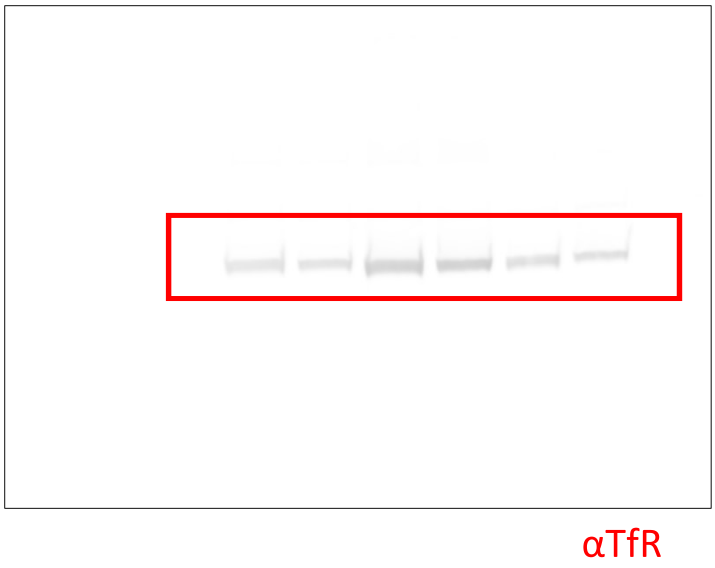

**Supplementary Figure S10**

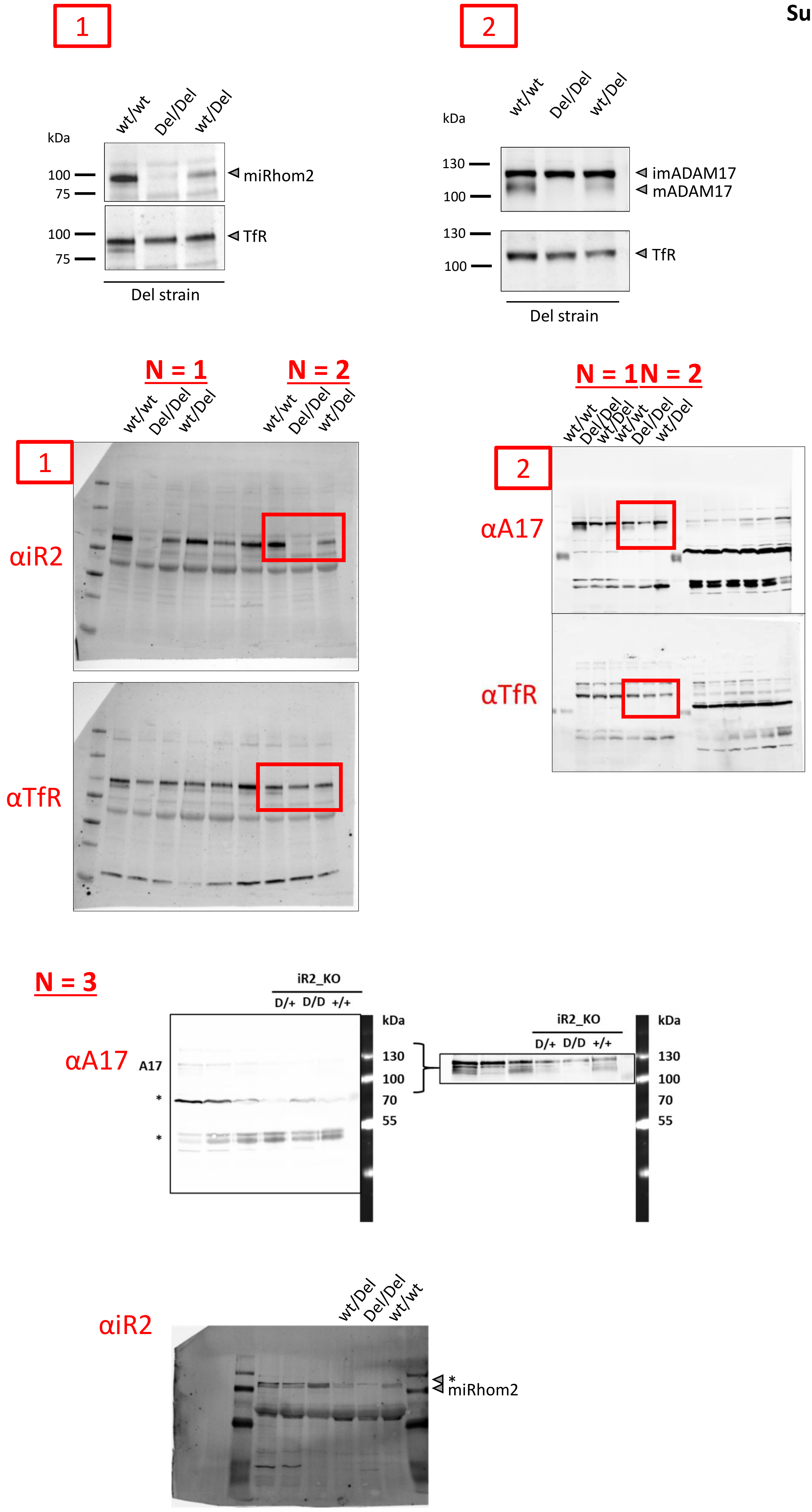

Supplement: Supplementary file 13 — Supplementary file13 (PDF 3016 KB) [file 18_2021_3845_MOESM13_ESM.pdf]
